# First-Trimester mRNA COVID-19 Vaccination and Risk of Major Congenital Anomalies

**DOI:** 10.1001/jamanetworkopen.2025.38039

**Published:** 2025-10-15

**Authors:** Clément Bernard, Tom Duchemin, Lise Marty, Jérôme Drouin, Sara Miranda, Laura Semenzato, Jérémie Botton, Laurent Chouchana, Rosemary Dray-Spira, Alain Weill, Mahmoud Zureik

**Affiliations:** 1 EPI-PHARE Scientific Interest Group in Epidemiology of Health Products, French National Agency for Medicines and Health Products Safety (ANSM) and French National Health Insurance (CNAM), Saint-Denis, France; 2 Centre for Epidemiology and Population Health (CESP), Anti-Infective Evasion and Pharmacoepidemiology Team, Paris-Saclay University, INSERM U1018, Villejuif, France; 3 Université Paris-Saclay, Faculté de pharmacie, Orsay, France; 4 Regional Center of Pharmacovigilance, Department of Perinatal, Pediatric and Adult Pharmacology, Groupe Hospitalier-Universitaire AP-HP, Hôpital Cochin, Paris, France

## Abstract

**Importance:**

Although messenger RNA (mRNA) COVID-19 vaccines are widely recommended during pregnancy, data on their fetal safety during the teratogenic window remain limited.

**Objective:**

To evaluate the association between first-trimester exposure to mRNA COVID-19 vaccines and the risk of major congenital malformations (MCMs), across 75 individual MCMs grouped by 13 organ systems.

**Design, Setting, and Participants:**

This nationwide, population-based cohort study was conducted using the comprehensive Mother-Child EPI-MERES Register, including all live-born infants in France from pregnancies starting April 1, 2021, to January 31, 2022. Follow-up data were available up to December 2024.

**Exposure:**

Exposure was defined as receiving at least 1 dose of mRNA-based COVID-19 vaccine during the first trimester of pregnancy.

**Main Outcomes and Measures:**

MCMs were identified following European Surveillance of Congenital Anomalies guidelines. The main analyses compared exposed and nonexposed infants, and sensitivity analyses used alternative comparison groups based on maternal vaccination status and timing. Confounders were adjusted for using a propensity score–based standardized mortality ratio weighting method. Weighted odds ratios (ORs) were estimated using logistic regression.

**Results:**

Among 527 564 eligible live-born infants, 130 338 (24.7%) were exposed to at least 1 vaccine dose during the first trimester. Mothers of exposed infants were slightly older than mothers of unexposed infants (mean [SD] age, 30.4 [5.3] years vs 30.1 [5.1] years), were less socially deprived, and presented slightly more often with a comorbidity. The prevalence of MCMs was 176.6 per 10 000 (2302 infants) among exposed infants and 179.4 per 10 000 (7128 infants) among nonexposed infants. No increased risk of MCMs was observed overall (weighted OR, 0.98 [95% CI, 0.93-1.04]), by organ system (weighted ORs ranging from 0.84 [95% CI, 0.68-1.04] for digestive system MCMs to 1.20 [95% CI, 0.75-1.91] for abdominal wall defects), or for any of the 75 individual MCMs. Sensitivity and stratified analyses confirmed these findings.

**Conclusions and Relevance:**

In this cohort study of pregnancies exposed to mRNA COVID-19 vaccines in the first trimester, exposure was not associated with an increased risk of any MCMs, overall, by organ group, or by individual MCM, supporting the safety of mRNA COVID-19 vaccines in early pregnancy.

## Key Points

**Question:**

Are messenger RNA (mRNA)–based COVID-19 vaccines teratogenic ?

**Findings:**

In this nationwide cohort study of 527 564 live-born infants, 130 338 (24.7%) were exposed to an mRNA-based COVID-19 vaccine during the first trimester of pregnancy. There was no association with an increased risk for 75 different major congenital malformations, whether examined overall, grouped by organ systems, or individually.

**Meaning:**

These findings suggest that mRNA-based COVID-19 vaccines do not appear to have any teratogenic effects.

## Teaser

This cohort study examines the association between first-trimester exposure to messenger RNA (mRNA) COVID-19 vaccines and the risk of major congenital malformations among infants in France.

## Introduction

Numerous studies have suggested that pregnant women are at greater risk of a severe COVID-19 infection compared with nonpregnant women of the same age,^1,2^ because of physiological and immunological changes during pregnancy. When women are infected during pregnancy, the risks of complications increase significantly,^3,4,5,6,7^ particularly for preterm birth^3,4,5^ and both maternal and infant morbidity and mortality.^5,6,8^ To prevent these adverse outcomes, messenger RNA (mRNA)–based COVID-19 vaccines have widely been recommended during pregnancy,^9^ initially only in high-income countries and subsequently worldwide.^10^

In early studies, and later in more comprehensive ones, these mRNA COVID-19 vaccines were shown to be highly effective in protecting pregnant women against severe infection during pregnancy^11,12,13,14^ and in providing immunity to infants during the first few months of life.^15,16,17,18^ Multiple nationwide studies reported no increase in risks of complications or adverse pregnancy outcomes following COVID-19 vaccination,^19,20,21,22,23^ especially no increase in the risk of stillbirth^19,20,21,22,23^ or miscarriage.^12,24,25^

Major congenital malformations (MCMs) are rare events, affecting 2% to 3% of births overall according to the European malformation surveillance program European Surveillance of Congenital Anomalies (EUROCAT).^26,27^ Each MCM has a complex cause involving a mix of genetic, environmental, medical, and unknown risk factors.^28^ The relevant drug or vaccine exposure window for studying an association with these outcomes is during organogenesis, which occurs in the first trimester of pregnancy, with a particularly sensitive window during the first 8 gestational weeks.

To date, only 2 large-scale studies have assessed the risk of MCMs, grouped by organ systems, resulting from in utero exposure to mRNA COVID-19 vaccines during the first trimester of pregnancy. The first study^29^ utilized a Scandinavian birth register and included 29 135 exposed newborns from Norway, Sweden, and Denmark. That study assessed associations for 11 of the 13 groups of MCMs as classified by EUROCAT and found no statistically significant increased risk, with adjusted odds ratios (ORs) ranging from 0.44 (95% CI, 0.18-1.05) for ear, face, and neck anomalies to 1.69 (95% CI, 0.76-3.78) for abdominal wall anomalies. The second study,^30^ based on the Ontario, Canada, birth register, included 34 181 exposed newborns and assessed associations for 8 organ system groups. It also found no increase in the risk of MCM for any of the study groups.

Both studies may have limited statistical power for rare MCMs such as ear, face, and neck anomalies, respiratory anomalies, and abdominal wall defects, resulting in wide 95% CIs in the Scandinavian study^29^ and no estimates at all in the Canadian study.^30^ Furthermore, neither study estimated the association with the risk of individual MCMs following first-trimester exposure to mRNA COVID-19 vaccines, which is crucial to the evaluation of the dilution of risks that could potentially be masking other factors associated with specific MCMs. On the basis of a previous study of vaccination rates among pregnant women in France,^31^ we estimate that the number of women exposed in the first trimester in France is approximately 150 000. Given that mRNA technology represents an important future of vaccines^32,33^ and is expected to be widely used, understanding its fetal safety profile is essential. Thus, the objective of this study was to evaluate the risk of MCMs following first-trimester exposure to an mRNA-based COVID-19 vaccine, first overall, second by organ system group, and then third by individual MCM.

## Methods

### Data Source

This nationwide cohort study used data from the Mother-Child EPI-MERES Register, which is nested within the French national Health Data System (SNDS). The SNDS covers over 99% of the French population, approximately 67 million people, whereby each individual is assigned a unique and pseudonymized identifier. Since 2006, the SNDS has recorded all reimbursement data for both outpatient care (including drug dispensation, outpatient visits, and laboratory test) and inpatient care (including diagnoses and procedures performed), as well as fully reimbursed health expenditures for patients with long-term diseases, such as cancer and diabetes. All individuals have a lifelong social security number, which is needed every time an individual seeks health care services. The reimbursement procedure is automated through the social security card. In addition, hospital data are collected, validated, and transmitted to paying and controlling institutions by physicians working in private and public hospitals. Therefore, data used in this study are exhaustive and can be considered accurate.^34^ In addition, the SNDS includes a COVID-19 vaccination database, VAC-SI, which records data at the individual dose level, including the recipient’s identifier, administration date, and the vaccine’s type. This database was used to conduct major nationwide efficacy and safety studies of the COVID-19 vaccines in France.^35,36,37,38,39^ The EPI-PHARE research group has permanent regulatory access to the anonymized data from the French National Health Data System (French decree No. 2016-1871, French law articles Art. R. 1461-13/14, French data protection authority decision CNIL-2016-316); thus, informed consent was not needed from patients. This study was registered in the EPI-PHARE SNDS-based registry (reference T 2024 10 436) and did not require prior institutional review board approval. This study followed the Strengthening the Reporting of Observational Studies in Epidemiology (STROBE) reporting guidelines.

The EPI-MERES Register includes all pregnancies that ended in France since January 1, 2010, was developed by the EPI-PHARE team for perinatal pharmacoepidemiology studies, links pregnancy data with infant data, and provides the last menstrual period date (LMP), conception date, pregnancy outcome date, gestational age, sociodemographic characteristics, indicators of comorbidities, and lifestyle factors such as tobacco or alcohol consumption. It also includes pregnancy-related information, including parity, folic acid consumption indicators, routine pregnancy ultrasonography, and teratogenic infection or drug consumption records. These variables are derived from SNDS data, primarily using *International Statistical Classification of Diseases and Related Health Problems, Tenth Revision (ICD-10)* diagnostic codes, Classification Commune des Actes Médicaux procedure codes, and records of dispensed medications. The EPI-MERES Register has been extensively described and used in previous studies to investigate drug safety during pregnancy.^40,41,42,43,44,45^

In this study, we included all live-born infants from pregnancies that began between April 1, 2021, and January 31, 2022, to mothers aged 15 to 49 years at conception, residing in mainland France (552 992 mothers). The date of conception was calculated using the pregnancy outcome date and the gestational age, or, when missing (0.4%), the date of the LMP entered by the physician at the end of the pregnancy. Same-sex twins were excluded as we cannot distinguish between the twins in the SNDS (11 399 infants [2.1%]). Infants whose mothers experienced teratogenic infections (2254 mothers [0.4%]) or consumed teratogenic drugs during pregnancy (529 mothers [0.1%]) were also excluded (the identification algorithms are detailed in eTable 1 in Supplement 1).

### Exposure and Control Groups

Vaccination against COVID-19 was recommended for pregnant women in France starting April 6, 2021,^31^ with mRNA COVID-19 vaccines being the only ones recommended for this population. The primary exposure to an mRNA COVID-19 vaccine during the first trimester was defined as receiving at least 1 dose of an mRNA COVID-19 vaccine between day 0 (date of conception) and day 91 (last day of the first trimester). Infants not meeting this criterion were considered unexposed. Since non-mRNA COVID-19 vaccines were never recommended for pregnant women in France,^31^ very few eligible women received such vaccines and were excluded from this study (11 226 women [2.1%]). Vaccines were available to the targeted population at every step of the vaccination campaign rollout, and we found no indication that there were any vaccine shortage.^31^

### Outcomes

According to the classification system of the EUROCAT guidelines,^46^ 75 MCMs were identified from the SNDS data.^43^ As described in eTable 2 in Supplement 1, the identification algorithms were based on inpatient *ICD-10* diagnostic codes and on relevant surgery or medical procedures (if any) in the records of live-born infants within 1 year after delivery for 72 of the 75 MCMs, and up to 2 years for 3 others: epispadias, hypospadias, and microcephaly. The follow-up data were available up to December 2024. All MCMs were then grouped into 13 organ systems: cardiovascular; nervous system; eye; ear, face, and neck; respiratory system; orofacial clefts; digestive system; abdominal wall; urinary system; genital; limb; chromosomal; and other anomalies.

### Statistical Analysis

For the main analysis, all nonexposed infants served as the control group. We estimated ORs for the overall risk of MCMs and for 13 groups of MCMs categorized by organ systems and then for each 1 of the 75 individual MCMs, using logistic regression models with propensity score–based standardized mortality ratio weighting^47,48^ to control for confounding factors. This method reweights the nonexposed group to match the distribution of observed covariates in the exposed group, thus providing covariate balance and allowing estimation of the average treatment effect among the exposed. This approach is particularly suited to our study objective and population, although the effect estimate primarily reflects the exposed group, which may limit generalizability if that group is highly unrepresentative of the overall population. The weights were derived from a propensity score representing the estimated probability of receiving at least 1 COVID-19 vaccine dose during the first trimester of pregnancy, estimated using a multivariate logistic regression. This model included various covariates related to maternal demographics, socioeconomic status, health behaviors, and preexisting medical conditions. These variables were all used and detailed in previous studies.^31,44,45,49^ Observations with nonoverlapping propensity scores between groups were removed for all association estimations (1 and 12 observations were removed in the main exposed and control groups, respectively). We evaluated the balance of covariate distributions using standardized mean differences (SMDs). We addressed missingness as follows: for the majority of variables, similar to most studies based on medico-administrative data, the absence of data was considered as the absence of the measured event (eg, no reimbursement for cancer treatment and/or hospitalization interpreted as absence of cancer). For the quintile of the social deprivation index of the city of residence, we created an unknown category to account for participants living in areas where a social deprivation index could not be computed (eg, villages with extremely small population size). Women with missing region of residence (15 women) were not included in this study. To evaluate any interaction with important factors, the main analysis by organ system group was stratified by maternal age at conception (≥35 years vs <35 years), quintiles of the social deprivation index of the city of residence^50^ (quintiles 1 and 2 vs quintiles 3-5), and dispensation of folic acid supplementation during the periconception period (yes vs no).

Sensitivity analyses were also conducted on the results for overall and by organ system groups. First, we compared the main exposed group to each of these 3 exclusive subgroups of the main control group: a first control subgroup including all infants born to mothers who received at least 1 dose of vaccine during pregnancy but not during the first trimester (ie, during the second or third trimester), a second control subgroup where mothers received at least 1 dose before pregnancy (up to the conception date), and a third control subgroup where mothers did not receive any dose during pregnancy or prior to conception. In another sensitivity analysis, we excluded from the main control group infants born to mothers who received at least 1 vaccine dose 30 days or less before the estimated conception date to account for potential immunogenicity effects of the vaccine. In other sensitivity analyses, we successively excluded from the main exposed group infants exposed before 3 weeks’ gestation and after 10 weeks’ gestation to test specific exposure during the most sensitive window for organogenesis,^40^ restricted the main exposed group to those exposed in the context of a primary vaccination series (ie, first and/or second dose) vs those exposed in the context of a booster dose (ie, third dose), and restricted the main exposed group to those exposed to a single dose of vaccine vs those exposed to 2 doses of vaccine. In a supplementary sensitivity analysis, we used overlap weighting^48^ as an alternative propensity score–based weighting method, and in another one we excluded from both main exposure and control groups all infants born to women who had a polymerase chain reaction–confirmed COVID-19 infection since its potential teratogenic effects—although no study has yet estimated a precise effect—cannot be ruled out.^29,51^ Finally, since the study population was limited to live births, we estimated the crude risk ratio of stillbirths among pregnancies exposed to first-trimester mRNA COVID-19 compared with those unexposed. Data were analyzed with R statistical software version 4.3.3 (R Project for Statistical Computing). Statistical significance was defined as a 95% CI that did not include the null value of 1.

## Results

A total of 527 564 infants were included in this study, of whom 130 338 (24.7%) were exposed to an mRNA COVID-19 vaccine dose during the first trimester of pregnancy (Table 1). Baseline characteristics showed small but statistically significant differences between groups, with most SMDs less than 0.10 (eTable 3 in Supplement 1). Mothers of exposed infants were slightly older (mean [SD] age, 30.4 [5.3] years; 28 496 infants [21.8%] born to mothers aged ≥35 years) than mothers of unexposed infants (mean [SD] age, 30.1 [5.1] years; 82 151 infants [20.6%] born to mothers aged ≥35 years), were less socially deprived (23 916 exposed infants [18.3%] vs 80 685 unexposed infants [20.3%] lived in fifth-quintile cities), and presented slightly more often with comorbidities, including hypertension (1650 exposed infants [1.3%] vs 4373 unexposed infants [1.1%]), antidepressant use (4496 exposed infants [3.4%] vs 10 913 unexposed infants [2.7%]), and anxiolytic or hypnotic use (3236 exposed infants [2.5%] vs 8075 unexposed infants [2.0%]). After weighting, no significant differences between the 2 groups for baseline characteristics remained (all SMDs were null) (eTable 3 and eFigure 1 in Supplement 1).

**Table 1.** Maternal Characteristics of the Main Exposure and Control Groups

Characteristic	Infants, No. (%)	
	Nonexposed (n = 397 226 [75%])	Exposed to ≥1 dose of an mRNA COVID-19 vaccine during the first trimester of pregnancy (n = 130 338 [25%])
Sociodemographic		
Age group, y		
<20	8598 (2.2)	2209 (1.7)
20-24	51 338 (12.9)	14 422 (11.1)
25-29	121 374 (30.6)	38 808 (29.8)
30-34	133 765 (33.7)	46 403 (35.6)
35-39	66 929 (16.8)	23 379 (17.9)
≥40	15 222 (3.8)	5117 (3.9)
Social deprivation index quintiles		
First (least deprived)	78 177 (19.7)	28 280 (21.7)
Second	81 035 (20.4)	27 221 (20.9)
Third	78 226 (19.7)	25 384 (19.5)
Fourth	76 281 (19.2)	24 418 (18.7)
Fifth (most deprived)	80 685 (20.3)	23 916 (18.3)
Unknown	2822 (0.7)	1119 (0.9)
Complementary health coverage (C2S)	68 498 (17.2)	15 529 (11.9)
State medical aid (AME)	1937 (0.5)	158 (0.1)
Region of residence		
Auvergne Rhône Alpes	49 955 (12.6)	15 860 (12.2)
Bourgogne Franche Comte	14 725 (3.7)	4975 (3.8)
Bretagne	17 028 (4.3)	7342 (5.6)
Centre Val De Loire	14 726 (3.7)	5104 (3.9)
Corse	1633 (0.4)	372 (0.3)
Grand Est	30 308 (7.6)	9537 (7.3)
Hauts de France	37 008 (9.3)	13 616 (10.4)
Ile de France	91 968 (23.2)	27 188 (20.9)
Normandie	18 541 (4.7)	7564 (5.8)
Nouvelle Aquitaine	30 518 (7.7)	11 441 (8.8)
Occitanie	34 930 (8.8)	10 022 (7.7)
Pays de la Loire	21 488 (5.4)	9167 (7.0)
Provence Alpes Côte d’Azur	34 398 (8.7)	8150 (6.3)
Pregnancy monitoring		
Ultrasonography		
First trimester	353 678 (89.0)	119 179 (91.4)
Second trimester	369 380 (93.0)	123 198 (94.5)
Third trimester	371 785 (93.6)	123 376 (94.7)
Periconception folic acid dispensation	212 477 (53.5)	73 981 (56.8)
No. of pregnancies <22 wk (since 2006)		
0	297 580 (74.9)	98 988 (75.9)
1	70 494 (17.7)	22 664 (17.4)
≥2	29 152 (7.3)	8686 (6.7)
No. of pregnancies >22 wk (since 2006)		
First pregnancy	148 582 (37.4)	49 029 (37.6)
Second pregnancy	125 271 (31.5)	43 247 (33.2)
Third pregnancy and more	123 373 (31.1)	38 062 (29.2)
Use of assisted reproductive technology	14 515 (3.7)	4411 (3.4)
Confirmed COVID-19 infection during the first trimester of pregnancy	28 213 (7.1)	4966 (3.8)
Lifestyle habits		
Tobacco consumption	46 753 (11.8)	16 125 (12.4)
Alcohol consumption	2153 (0.5)	829 (0.6)
Opioid consumption	2083 (0.5)	616 (0.5)
Comorbidities		
Obesity	27 805 (7.0)	9723 (7.5)
Preexisting diabetes	3342 (0.8)	1294 (1.0)
Hypertension	4373 (1.1)	1650 (1.3)
Stroke	480 (0.1)	175 (0.1)
Other cardiovascular disease	2583 (0.7)	921 (0.7)
Antidepressant use	10 913 (2.7)	4496 (3.4)
Neuroleptic use	1692 (0.4)	715 (0.5)
Anxiolytic or hypnotic use	8075 (2.0)	3236 (2.5)
Multiple sclerosis	736 (0.2)	265 (0.2)
Epilepsy	1097 (0.3)	422 (0.3)
Chronic respiratory disease	9554 (2.4)	3541 (2.7)
Inflammatory or skin disease	10 407 (2.6)	3576 (2.7)
Cancer	2982 (0.8)	1013 (0.8)
HIV infection	581 (0.1)	242 (0.2)

Abbreviations: AME, Aide Médicale d'État; C2S, Complémentaire Santé Solidarité; mRNA, messenger RNA.

Overall, we observed 2302 infants with MCMs in the exposed group (rate, 176.6 MCMs per 10 000) and 7128 infants with MCMs in the control group (rate, 179.4 MCMs per 10 000), yielding a nonsignificant weighted OR of 0.98 (95% CI, 0.93-1.04). No association was observed when analyzing MCMs by organ system, with weighted ORs ranging from 0.84 (95% CI, 0.68-1.04) for digestive system malformations to 1.20 (95% CI, 0.75-1.91) for abdominal wall defects. Among all 13 organ system–specific risk estimates, 10 had weighted ORs below 1.10 (Table 2).

**Table 2.** Associations Between a First-Trimester Exposure to mRNA-Based COVID-19 Vaccines and the Risk of MCMs

Type of MCM	No. of infants (No. of MCMs/10 000)		OR (95% CI)	
	Exposed (n = 130 337)	Control (n = 397 214	Unweighted	SMR weighted
Any MCM	2302 (176.6)	7128 (179.4)	0.98 (0.94-1.03)	0.98 (0.93-1.04)
Cardiac MCM	760 (58.3)	2315 (58.3)	1.00 (0.92-1.09)	1.01 (0.92-1.12)
Truncus arteriosus	7 (0.5)	10 (0.3)	2.13 (0.81-5.60)	2.63 (0.64-10.81)
Double outlet right ventricle	11 (0.8)	45 (1.1)	0.74 (0.39-1.44)	0.84 (0.38-1.87)
Single ventricle	12 (0.9)	25 (0.6)	1.46 (0.73-2.91)	1.48 (0.61-3.61)
Ventricular septal defect	350 (26.9)	1067 (26.9)	1.00 (0.89-1.13)	1.00 (0.86-1.16)
Atrial septal defect	335 (25.7)	1028 (25.9)	0.99 (0.88-1.12)	1.00 (0.86-1.16)
Atrioventricular septal defect	34 (2.6)	143 (3.6)	0.72 (0.50-1.05)	0.75 (0.48-1.18)
Tetralogy of Fallot	43 (3.3)	125 (3.1)	1.05 (0.74-1.48)	1.09 (0.71-1.67)
Mitral stenosis	2 (0.2)	8 (0.2)	0.76 (0.16-3.59)	0.81 (0.13-5.18)
Ebstein anomaly	4 (0.3)	13 (0.3)	0.94 (0.31-2.88)	0.99 (0.25-3.94)
Pulmonary valve stenosis	52 (4.0)	152 (3.8)	1.04 (0.76-1.43)	1.02 (0.69-1.50)
Pulmonary valve atresia	16 (1.2)	45 (1.1)	1.08 (0.61-1.92)	1.11 (0.54-2.26)
Aortic valve stenosis	8 (0.6)	33 (0.8)	0.74 (0.34-1.60)	0.76 (0.30-1.90)
Tricuspid atresia	8 (0.6)	13 (0.3)	1.88 (0.78-4.53)	2.08 (0.62-7.03)
Hypoplastic left heart syndrome	15 (1.2)	54 (1.4)	0.85 (0.48-1.50)	0.90 (0.45-1.82)
Hypoplastic right heart syndrome	6 (0.5)	23 (0.6)	0.80 (0.32-1.95)	0.94 (0.31-2.86)
Coarctation of the aorta	44 (3.4)	125 (3.1)	1.07 (0.76-1.51)	1.11 (0.72-1.70)
Aortic atresia	9 (0.7)	17 (0.4)	1.61 (0.72-3.62)	1.84 (0.61-5.52)
Total anomalous pulmonary venous return	10 (0.8)	15 (0.4)	2.03 (0.91-4.52)	2.23 (0.73-6.77)
Patent ductus arteriosus	19 (1.5)	58 (1.5)	1.00 (0.59-1.68)	1.06 (0.55-2.01)
Corrected transposition of the great arteries	1 (0.1)	3 (0.1)	1.02 (0.11-9.77)	1.00 (0.06-16.02)
Transposition of the great arteries	42 (3.2)	142 (3.6)	0.90 (0.64-1.27)	0.89 (0.59-1.34)
Limb MCM	421 (32.3)	1188 (29.9)	1.08 (0.97-1.21)	1.09 (0.95-1.25)
Limb shortening	34 (2.6)	86 (2.2)	1.20 (0.81-1.79)	1.16 (0.71-1.89)
Clubfoot	120 (9.2)	347 (8.7)	1.05 (0.86-1.30)	1.07 (0.83-1.38)
Hip dislocation	101 (7.7)	229 (5.8)	1.34 (1.06-1.70)	1.23 (0.92-1.64)
Polydactyly	153 (11.7)	504 (12.7)	0.93 (0.77-1.11)	0.98 (0.78-1.22)
Syndactyly	19 (1.5)	35 (0.9)	1.65 (0.95-2.89)	1.71 (0.82-3.60)
Kidney or urinary tract MCM	304 (23.3)	1018 (25.6)	0.91 (0.80-1.03)	0.88 (0.76-1.03)
Unilateral renal agenesis	65 (5.0)	194 (4.9)	1.02 (0.77-1.35)	0.96 (0.68-1.35)
Bilateral renal agenesis	2 (0.2)	8 (0.2)	0.76 (0.16-3.59)	0.78 (0.12-4.99)
Renal dysplasia	29 (2.2)	117 (2.9)	0.76 (0.50-1.13)	0.76 (0.47-1.24)
Hydronephrosis	171 (13.1)	552 (13.9)	0.94 (0.80-1.12)	0.91 (0.74-1.12)
Lobulated or ectopic kidney	29 (2.2)	144 (3.6)	0.61 (0.41-0.91)	0.59 (0.37-0.94)
Epispadias exstrophy of the bladder	5 (0.4)	7 (0.2)	2.18 (0.69-6.86)	2.37 (0.47-11.86)
Posterior urethral valves	17 (1.3)	50 (1.3)	1.04 (0.60-1.80)	0.99 (0.50-1.93)
Genital MCM	242 (18.6)	791 (19.9)	0.93 (0.81-1.08)	0.91 (0.77-1.09)
Hypospadias	233 (17.9)	766 (19.3)	0.93 (0.80-1.07)	0.91 (0.76-1.08)
Indeterminate sex	9 (0.7)	30 (0.8)	0.91 (0.43-1.93)	0.97 (0.39-2.43)
Digestive system MCM	151 (11.6)	551 (13.9)	0.83 (0.70-1.00)	0.84 (0.68-1.04)
Esophageal atresia	33 (2.5)	130 (3.3)	0.77 (0.53-1.13)	0.76 (0.49-1.20)
Duodenal atresia	17 (1.3)	48 (1.2)	1.08 (0.62-1.88)	1.11 (0.55-2.20)
Atresia of other parts of the small intestine	10 (0.8)	45 (1.1)	0.68 (0.34-1.34)	0.69 (0.31-1.54)
Anorectal atresia	42 (3.2)	145 (3.7)	0.88 (0.63-1.24)	0.92 (0.60-1.39)
Hirschsprung disease	14 (1.1)	53 (1.3)	0.81 (0.45-1.45)	0.82 (0.41-1.67)
Intestinal malrotation	8 (0.6)	43 (1.1)	0.57 (0.27-1.21)	0.56 (0.23-1.32)
Biliary atresia	5 (0.4)	17 (0.4)	0.90 (0.33-2.43)	0.95 (0.28-3.24)
Annular pancreas	1 (0.1)	3 (0.1)	1.02 (0.11-9.77)	1.56 (0.07-35.90)
Diaphragmatic hernia	29 (2.2)	94 (2.4)	0.94 (0.62-1.43)	0.92 (0.56-1.53)
Cleft lip or palate	169 (13.0)	505 (12.7)	1.02 (0.86-1.21)	1.00 (0.81-1.24)
Cleft palate	55 (4.2)	163 (4.1)	1.03 (0.76-1.40)	1.04 (0.71-1.52)
Cleft lip or cleft lip and palate	114 (8.7)	342 (8.6)	1.02 (0.82-1.26)	0.98 (0.76-1.28)
Nervous system MCM	161 (12.4)	499 (12.6)	0.98 (0.82-1.17)	1.04 (0.83-1.29)
Encephalocele	2 (0.2)	17 (0.4)	0.36 (0.08-1.55)	0.39 (0.08-2.01)
Spina bifida	19 (1.5)	51 (1.3)	1.14 (0.67-1.92)	1.15 (0.59-2.22)
Hydrocephaly	24 (1.8)	84 (2.1)	0.87 (0.55-1.37)	0.89 (0.51-1.54)
Microcephaly	70 (5.4)	227 (5.7)	0.94 (0.72-1.23)	1.01 (0.73-1.41)
Arhinencephaly holoprosencephaly	3 (0.2)	6 (0.2)	1.52 (0.38-6.09)	1.98 (0.28-13.95)
Malformation of the corpus callosum	48 (3.7)	123 (3.1)	1.19 (0.85-1.66)	1.25 (0.82-1.91)
Chromosomal anomaly	93 (7.1)	359 (9.0)	0.79 (0.63-0.99)	0.85 (0.65-1.12)
Down syndrome	66 (5.1)	261 (6.6)	0.77 (0.59-1.01)	0.85 (0.61-1.18)
Trisomy 13 (Patau syndrome)	2 (0.2)	13 (0.3)	0.47 (0.11-2.08)	0.60 (0.10-3.43)
Trisomy 18 (Edwards syndrome)	3 (0.2)	29 (0.7)	0.32 (0.10-1.03)	0.34 (0.09-1.28)
Turner syndrome	7 (0.5)	16 (0.4)	1.33 (0.55-3.24)	1.26 (0.41-3.85)
Skeletal dysplasia	15 (1.2)	40 (1.0)	1.14 (0.63-2.07)	1.09 (0.52-2.26)
Eye MCM	42 (3.2)	114 (2.9)	1.12 (0.79-1.60)	1.17 (0.75-1.83)
Anophthalmia or microphthalmia	7 (0.5)	32 (0.8)	0.67 (0.29-1.51)	0.72 (0.27-1.90)
Congenital cataract	22 (1.7)	66 (1.7)	1.02 (0.63-1.65)	1.00 (0.55-1.81)
Congenital glaucoma	13 (1.0)	30 (0.8)	1.32 (0.69-2.53)	1.46 (0.62-3.41)
Abdominal wall MCM	39 (3.0)	100 (2.5)	1.19 (0.82-1.72)	1.20 (0.75-1.91)
Gastroschisis	19 (1.5)	55 (1.4)	1.05 (0.62-1.77)	1.14 (0.59-2.21)
Omphalocele	20 (1.5)	48 (1.2)	1.27 (0.75-2.14)	1.18 (0.62-2.25)
Respiratory system MCM	6 (0.5)	18 (0.5)	1.02 (0.40-2.56)	1.00 (0.32-3.12)
Choanal atresia	6 (0.5)	18 (0.5)	1.02 (0.40-2.56)	1.00 (0.32-3.12)
Ear, face, or neck MCM	5 (0.4)	12 (0.3)	1.27 (0.45-3.60)	1.14 (0.32-4.09)
Anotia	5 (0.4)	12 (0.3)	1.27 (0.45-3.60)	1.14 (0.32-4.09)
Other MCM	123 (9.4)	427 (10.7)	0.88 (0.72-1.07)	0.89 (0.70-1.14)
Craniosynostosis	58 (4.5)	181 (4.6)	0.98 (0.73-1.31)	0.97 (0.68-1.39)
Situs inversus	3 (0.2)	36 (0.9)	0.25 (0.08-0.82)	0.25 (0.07-0.88)
Dysplasia of the septum of the optic pathways	3 (0.2)	18 (0.5)	0.51 (0.15-1.72)	0.61 (0.15-2.58)
Vascular lesions	42 (3.2)	143 (3.6)	0.90 (0.63-1.26)	0.95 (0.62-1.45)
Laterality anomalies	13 (1.0)	65 (1.6)	0.61 (0.34-1.11)	0.61 (0.31-1.22)
Other malformation	7 (0.5)	21 (0.5)	1.02 (0.43-2.39)	0.90 (0.32-2.49)

Abbreviations: MCM, major congenital malformation; mRNA, messenger RNA; OR, odds ratio; SMR, standardized mortality ratio.

This study found no statistically significant increased risks for any of the 75 individual MCMs associated with first-trimester exposure to mRNA COVID-19 vaccines, with weighted ORs of 1.00 (95% CI, 0.86-1.16) for ventricular septal defect, 1.00 (95% CI, 0.86-1.16) for atrial septal defect, and 0.91 (95% CI, 0.74-1.12) for hydronephrosis. Slight reductions in risk were observed for 2 MCMs: lobulated or ectopic kidney (weighted OR, 0.59; 95% CI, 0.37-0.94) and situs inversus (weighted OR, 0.25; 95% CI, 0.07-0.88). Four weighted ORs exceeded 2-fold, although they were not statistically significant: 3 cardiovascular system MCMs—truncus arteriosus (weighted OR, 2.63; 95% CI, 0.64-10.81), tricuspid atresia (weighted OR, 2.08; 95% CI, 0.62-7.03), and total anomalous pulmonary venous return (weighted OR, 2.23; 95% CI, 0.73-6.77)—and 1 urinary system MCM, epispadias and/or exstrophy of the bladder (weighted OR, 2.37; 95% CI, 0.47-11.86). For 5 MCMs (double outlet left ventricle, anencephaly, triploidy and/or polyploidy, anophthalmia, and prune belly syndrome), no cases were reported in the exposed group or the control group, making risk estimation impossible for these MCMs. Among the 64 remaining estimations, 17 were greater than 1.10 and 22 were less than 0.90, but none was statistically significant. When stratifying by age, social deprivation, and folic acid consumption, we did not find an underlying increased risk for any of the 13 organ system groups across any strata (Table 3).

**Table 3.** Organ System Groups Results Stratified by Age, Social Deprivation, and Folic Acid Consumption

Type of MCM and analysis group	No. of infants/total No. of infants in the group (No. of MCMs/10 000)		Weighted OR (95% CI)
	Exposed	Control	
Any MCM			
Main analysis	2302/130 337 (176.6)	7128/397 214 (179.4)	0.98 (0.93-1.04)
Age, y			
<35	1779/101 839 (174.7)	5441/315 065 (172.7)	1.01 (0.95-1.08)
≥35	521/28 495 (182.8)	1687/82 129 (205.4)	0.89 (0.79-1.01)
Social deprivation index			
Most deprived (third to fifth quintile)	1404/74 837 (187.6)	4359/238 003 (183.1)	1.02 (0.95-1.10)
Least deprived (first and second quintile)	897/55 496 (161.6)	2768/159 203 (173.9)	0.93 (0.85-1.02)
Folic acid consumption			
No	1004/56 357 (178.2)	3299/184 734 (178.6)	0.99 (0.91-1.08)
Yes	1298/73 980 (175.5)	3829/212 463 (180.2)	0.98 (0.91-1.06)
Cardiac MCM			
Main analysis	760/130 337 (58.3)	2315/397 214 (58.3)	1.01 (0.92-1.12)
Age, y			
<35	568/101 839 (55.8)	1737/315 065 (55.1)	1.03 (0.92-1.16)
≥35	191/28 495 (67.0)	578/82 129 (70.4)	0.96 (0.79-1.17)
Social deprivation index			
Most deprived (third to fifth quintile)	467/74 837 (62.4)	1453/238 003 (61.0)	1.04 (0.91-1.18)
Least deprived (first and second quintile)	292/55 496 (52.6)	862/159 203 (54.1)	0.97 (0.83-1.14)
Folic acid consumption			
No	339/56 357 (60.2)	1085/184 734 (58.7)	1.03 (0.89-1.20)
Yes	421/73 980 (56.9)	1230/212 463 (57.9)	0.99 (0.87-1.14)
Limb MCM			
Main analysis	421/130 337 (32.3)	1188/397 214 (29.9)	1.09 (0.95-1.25)
Age, y			
<35	336/101 839 (33.0)	934/315 065 (29.6)	1.13 (0.96-1.32)
≥35	85/28 495 (29.8)	254/82 129 (30.9)	0.96 (0.71-1.29)
Social deprivation index			
Most deprived (third to fifth quintile)	257/74 837 (34.3)	714/238 003 (30.0)	1.15 (0.96-1.37)
Least deprived (first and second quintile)	164/55 496 (29.6)	474/159 203 (29.8)	1.01 (0.81-1.25)
Folic acid consumption			
No	174/56 357 (30.9)	545/184 734 (29.5)	1.05 (0.85-1.31)
Yes	247/73 980 (33.4)	643/212 463 (30.3)	1.11 (0.93-1.33)
Kidney or urinary tract MCM			
Main analysis	304/130 337 (23.3)	1018/397 214 (25.6)	0.88 (0.76-1.03)
Age, y			
<35	237/101 839 (23.3)	806/315 065 (25.6)	0.88 (0.74-1.05)
≥35	67/28 495 (23.5)	212/82 129 (25.8)	0.89 (0.64-1.24)
Social deprivation index			
Most deprived (third to fifth quintile)	180/74 837 (24.1)	585/238 003 (24.6)	0.94 (0.77-1.15)
Least deprived (first and second quintile)	124/55 496 (22.3)	433/159 203 (27.2)	0.81 (0.64-1.03)
Folic acid consumption			
No	132/56 357 (23.4)	453/184 734 (24.5)	0.92 (0.73-1.16)
Yes	172/73 980 (23.2)	565/212 463 (26.6)	0.85 (0.69-1.04)
Genital MCM			
Main analysis	242/130 337 (18.6)	791/397 214 (19.9)	0.91 (0.77-1.09)
Age, y			
<35	198/101 839 (19.4)	622/315 065 (19.7)	0.97 (0.79-1.17)
≥35	44/28 495 (15.4)	169/82 129 (20.6)	0.74 (0.50-1.09)
Social deprivation index			
Most deprived (third to fifth quintile)	157/74 837 (21.0)	473/238 003 (19.9)	1.04 (0.83-1.29)
Least deprived (first and second quintile)	85/55 496 (15.3)	318/159 203 (20.0)	0.75 (0.57-0.99)
Folic acid consumption			
No	111/56 357 (19.7)	326/184 734 (17.6)	1.09 (0.83-1.43)
Yes	131/73 980 (17.7)	465/212 463 (21.9)	0.81 (0.64-1.02)
Digestive system MCM			
Main analysis	151/130 337 (11.6)	551/397 214 (13.9)	0.84 (0.68-1.04)
Age, y			
<35	120/101 839 (11.8)	414/315 065 (13.1)	0.91 (0.71-1.16)
≥35	31/28 495 (10.9)	137/82 129 (16.7)	0.66 (0.42-1.04)
Social deprivation index			
Most deprived (third to fifth quintile)	83/74 837 (11.1)	327/238 003 (13.7)	0.79 (0.59-1.06)
Least deprived (first and second quintile)	68/55 496 (12.3)	223/159 203 (14.0)	0.92 (0.66-1.28)
Folic acid consumption			
No	63/56 357 (11.2)	264/184 734 (14.3)	0.76 (0.55-1.06)
Yes	88/73 980 (11.9)	287/212 463 (13.5)	0.91 (0.68-1.21)
Cleft lip or palate			
Main analysis	169/130 337 (13.0)	505/397 214 (12.7)	1.00 (0.81-1.24)
Age, y			
<35	132/101 839 (13.0)	396/315 065 (12.6)	1.01 (0.80-1.29)
≥35	37/28 495 (13.0)	109/82 129 (13.3)	0.96 (0.61-1.51)
Social deprivation index			
Most deprived (third to fifth quintile)	103/74 837 (13.8)	341/238 003 (14.3)	0.93 (0.71-1.22)
Least deprived (first and second quintile)	66/55 496 (11.9)	164/159 203 (10.3)	1.14 (0.80-1.63)
Folic acid consumption			
No	70/56 357 (12.4)	231/184 734 (12.5)	0.96 (0.69-1.33)
Yes	99/73 980 (13.4)	274/212 463 (12.9)	1.04 (0.78-1.37)
Nervous system MCM			
Main analysis	161/130 337 (12.4)	499/397 214 (12.6)	1.04 (0.83-1.29)
Age, y			
<35	121/101 839 (11.9)	392/315 065 (12.4)	1.01 (0.78-1.30)
≥35	39/28 495 (13.7)	107/82 129 (13.0)	1.11 (0.70-1.75)
Social deprivation index			
Most deprived (third to fifth quintile)	110/74 837 (14.7)	340/238 003 (14.3)	1.06 (0.81-1.39)
Least deprived (first and second quintile)	51/55 496 (9.2)	159/159 203 (10.0)	0.99 (0.67-1.45)
Folic acid consumption			
No	74/56 357 (13.1)	239/184 734 (12.9)	1.03 (0.74-1.42)
Yes	87/73 980 (11.8)	260/212 463 (12.2)	1.04 (0.77-1.40)
Chromosomal anomaly			
Main analysis	93/130 337 (7.1)	359/397 214 (9.0)	0.85 (0.65-1.12)
Age, y			
<35	48/101 839 (4.7)	146/315 065 (4.6)	1.05 (0.70-1.57)
≥35	45/28 495 (15.8)	213/82 129 (25.9)	0.71 (0.48-1.04)
Social deprivation index			
Most deprived (third to fifth quintile)	64/74 837 (8.6)	227/238 003 (9.5)	0.97 (0.69-1.37)
Least deprived (first and second quintile)	29/55 496 (5.2)	132/159 203 (8.3)	0.68 (0.42-1.09)
Folic acid consumption			
No	47/56 357 (8.3)	207/184 734 (11.2)	0.77 (0.53-1.12)
Yes	46/73 980 (6.2)	152/212 463 (7.2)	0.96 (0.64-1.45)
Eye MCM			
Main analysis	42/130 337 (3.2)	114/397 214 (2.9)	1.17 (0.75-1.83)
Age, y			
<35	32/101 839 (3.1)	83/315 065 (2.6)	1.20 (0.72-2.01)
≥35	10/28 495 (3.5)	31/82 129 (3.8)	1.08 (0.44-2.64)
Social deprivation index			
Most deprived (third to fifth quintile)	25/74 837 (3.3)	66/238 003 (2.8)	1.29 (0.71-2.33)
Least deprived (first and second quintile)	17/55 496 (3.1)	48/159 203 (3.0)	1.06 (0.54-2.10)
Folic acid consumption			
No	21/56 357 (3.7)	48/184 734 (2.6)	1.55 (0.78-3.06)
Yes	21/73 980 (2.8)	66/212 463 (3.1)	0.96 (0.53-1.75)
Abdominal wall MCM			
Main analysis	39/130 337 (3.0)	100/397 214 (2.5)	1.20 (0.75-1.91)
Age, y			
<35	31/101 839 (3.0)	78/315 065 (2.5)	1.22 (0.72-2.05)
≥35	8/28 495 (2.8)	22/82 129 (2.7)	1.15 (0.42-3.16)
Social deprivation index			
Most deprived (third to fifth quintile)	22/74 837 (2.9)	57/238 003 (2.4)	1.22 (0.66-2.28)
Least deprived (first and second quintile)	17/55 496 (3.1)	43/159 203 (2.7)	1.18 (0.59-2.38)
Folic acid consumption			
No	15/56 357 (2.7)	50/184 734 (2.7)	0.96 (0.47-1.95)
Yes	24/73 980 (3.2)	50/212 463 (2.4)	1.46 (0.78-2.73)
Respiratory system MCM			
Main analysis	6/130 337 (0.5)	18/397 214 (0.5)	1.00 (0.32-3.12)
Age, y			
<35	4/101 839 (0.4)	14/315 065 (0.4)	0.88 (0.23-3.37)
≥35	2/28 495 (0.7)	4/82 129 (0.5)	1.42 (0.16-12.22)
Social deprivation index			
Most deprived (third to fifth quintile)	3/74 837 (0.4)	11/238 003 (0.5)	0.81 (0.18-3.72)
Least deprived (first and second quintile)	3/55 496 (0.5)	7/159 203 (0.4)	1.34 (0.24-7.55)
Folic acid consumption			
No	1/56 357 (0.2)	7/184 734 (0.4)	0.54 (0.05-6.23)
Yes	5/73 980 (0.7)	11/212 463 (0.5)	1.21 (0.33-4.46)
Ear, face, or neck MCM			
Main analysis	5/130 337 (0.4)	12/397 214 (0.3)	1.14 (0.32-4.09)
Age, y			
<35	5/101 839 (0.5)	9/315 065 (0.3)	1.62 (0.39-6.70)
≥35	0/28 495 (0.0)	3/82 129 (0.4)	0.00 (0.00-infinity)
Social deprivation index			
Most deprived (third to fifth quintile)	3/74 837 (0.4)	7/238 003 (0.3)	1.14 (0.22-5.96)
Least deprived (first and second quintile)	2/55 496 (0.4)	5/159 203 (0.3)	1.08 (0.15-7.98)
Folic acid consumption			
No	3/56 357 (0.5)	4/184 734 (0.2)	1.84 (0.27-12.34)
Yes	2/73 980 (0.3)	8/212 463 (0.4)	0.71 (0.12-4.35)
Other MCM			
Main analysis	123/130 337 (9.4)	427/397 214 (10.7)	0.89 (0.70-1.14)
Age, y			
<35	106/101 839 (10.4)	328/315 065 (10.4)	1.05 (0.80-1.37)
≥35	17/28 495 (6.0)	99/82 129 (12.1)	0.48 (0.27-0.85)
Social deprivation index			
Most deprived (third to fifth quintile)	68/74 837 (9.1)	262/238 003 (11.0)	0.82 (0.60-1.14)
Least deprived (first and second quintile)	55/55 496 (9.9)	165/159 203 (10.4)	1.00 (0.69-1.46)
Folic acid consumption			
No	51/56 357 (9.0)	208/184 734 (11.3)	0.79 (0.55-1.15)
Yes	72/73 980 (9.7)	219/212 463 (10.3)	0.99 (0.72-1.37)

Abbreviations: MCM, major congenital malformation; OR, odds ratio.

In the sensitivity analyses, excluding infants exposed to a COVID-19 infection during the first trimester of pregnancy from the main exposure groups did not alter any associations (eFigure 2 in Supplement 1). When comparing our main exposure group with the 3 distinct control subgroups—infants whose mothers received at least 1 vaccine dose during the second or third trimester, those whose mothers received at least 1 dose before conception, and those whose mothers received no doses until the end of pregnancy (characteristics detailed in eTable 4 in Supplement 1)—we found no increased risk for any MCMs grouped by organ system. Using overlap weighting as an alternative propensity score–weighting method yielded results consistent with the standardized mortality ratio weighting method. Other sensitivity analyses conducted did not reveal any increased risk for any organ system group of MCM.

In the stillbirth rate analysis, we found that 556 stillbirths (0.4%) happened after exposure to a mRNA COVID-19 vaccine during the first trimester of the pregnancy and 1773 stillbirths (0.4%) happened in the control group. The risk ratio was 0.96 (95% CI, 0.87-1.05), which was not statistically significant.

## Discussion

This cohort study including 130 338 infants exposed to at least 1 dose of mRNA COVID-19 vaccine during the first trimester of pregnancy found no association with an increased risk for any of the MCMs examined, either individually, overall, or grouped by organ system. The results were consistent across all stratification and sensitivity analyses, providing substantial reassurance that mRNA COVID-19 vaccines are highly unlikely to present a teratogenic risk.

Our findings align with previous research^29,30^ showing no association between in utero exposure to mRNA COVID-19 vaccines and the risk for any specific organ system MCM group. The highest and lowest estimates for the MCM groups of interest in the 2 previous studies^29,30^ did not exactly match those of this study, suggesting there was no consistent trend in the estimated associations. The estimated 95% CIs, benefiting from the larger sample size, are centered around an OR of 1 and indicate no association with an exposure during the first trimester. We also found no significant increase in risk for any of the 75 analyzed individual MCMs. Another recent study analyzing individual MCMs, a US study using claims data, also found no association between a broader exposure window (from 14 days before the LMP to 20 weeks of gestation) and 18 individual MCMs,^52^ including 12 725 exposed infants (with an undisclosed number exposed only during the first trimester). However, that study noted nonsignificant increased estimands for the risk of holoprosencephaly (adjusted prevalence ratio, 3.56; 95% CI, 0.64-19.99) and the risk of anophthalmos or microphthalmos (adjusted prevalence ratio, 3.15; 95% CI, 0.78-12.69). In contrast, our results do not show markedly increased estimands for these 2 MCMs, suggesting that their observations may be due to chance rather than an actual association with these specific MCMs.

The main strength of this study lies in its uniquely large, unselected cohort of pregnancies, resulting in the largest sample to date of infants exposed to mRNA-based COVID-19 vaccines during the first trimester, nearly 4 times larger than the previously largest study.^30^ To our knowledge, it is the only study to date to have estimated the risk of 75 individual MCMs following a first-trimester exposure to these vaccines, essential for evaluating their teratogenicity as each MCM is associated with specific developmental mechanisms and critical periods of vulnerability.

Our study confirms the fetal safety of mRNA COVID-19 vaccines during pregnancy, showing no increased risk of MCMs and reassuring the millions of women worldwide who received these vaccines early in pregnancy. Policymakers and health care practitioners should update vaccination guidelines to ensure that pregnant women are adequately informed about the safety of these vaccines. These findings also provide valuable insight into the fetal safety of mRNA-based vaccines, which could inform future considerations for their use in pregnant women.

### Limitations

We acknowledge several methodological limitations when interpreting this study’s results. First, the analysis was restricted to live births owing to difficulties in appropriately identifying MCMs among stillbirths and terminated pregnancies on the basis of health care data, similar to other studies on congenital anomalies based on this type of data.^29,30,40,41,52^ However, since COVID-19 mRNA-based vaccines have been shown to have no association with miscarriage^12,24,25^ or stillbirth^19,20,21,22,23^ and since we found a nonsignificant risk ratio of 0.96 for the stillbirth analysis, any observed association for high mortality MCMs is unlikely to be biased.^53^ Second, some comorbidities, such as obesity, may be slightly underestimated when detected using SNDS data,^40^ and although there may be unmeasured confounders like teratogenic environmental factors or genetic predisposition, these factors are not expected to substantially bias our effect estimates, given the set of variables already included. Third, the overall rate of MCM in this study (179.4 per 10 000 live births) is slightly lower than the EUROCAT surveillance (204 per 10 000 live births in 2022),^27^ which can mainly be attributed to 2 factors: first, most infants born to mothers who had teratogenic infections or consumed teratogenic drugs were excluded, reducing the overall rate; and second, we only included 75 of the 104 MCMs described in the EUROCAT guidelines^46^ because the SNDS provides only 3-digit *ICD-10* codes and we chose not to analyze nonspecific MCM codes. Using this method, our detection rates of individual MCMs among live-born infants were generally comparable to the EUROCAT rates.^43^ The lack of statistical significance and wide 95% CIs could be due to the rarity of these MCMs, as indicated by the very small number of participants experiencing these MCMs. The comprehensiveness of our study is also limited by the 5 extremely rare MCMs for which we could not assess an association since no case were reported either in the exposed group or the control group over our study period. Finally, among the numerous statistical tests performed for analyzing each individual MCM (75 independent tests) and for the stratified analyses of the MCMs grouped into organ systems (6 strata for 13 organ system groups, thus resulting in 78 independent tests), we found only 6 statistically significant associations, all in the direction of a reduced risk and most likely attributable to type I errors (ie, spurious observations resulting from the large number of tests).

## Conclusions

In this cohort study of pregnancies with first-trimester exposure to mRNA COVID-19 vaccines, exposure was not associated with an increased risk of MCMs in infants, whether grouped by organ system or considered individually. Although associations with extremely rare outcomes cannot be ruled out, these findings provide reassuring evidence regarding the safety of mRNA COVID-19 vaccination during early pregnancy.

## Notes

Supplement 1. eTable 1. Teratogenic infections and drug use identification algorithms eTable 2. Major congenital malformations (MCMs) detection algorithms eTable 3. Maternal characteristics table with standardized mean differences (SMDs) before and after standardized mortality ratio (SMR) weighting eFigure 1. Propensity score density for the primary exposed and control groups, before (left) and after SMR weighting (right) eTable 4. Maternal and pregnancy characteristics of children depending on whether they were exposed to at least one vaccine dose during the first trimester of pregnancy (G1), or the mother received at least one vaccine dose during the second or third trimester of pregnancy (G2), or the mother received at least one dose before conception (G3), or the mother received no doses until the end of pregnancy (G4) eFigure 2. Sensitivity analyses on the main results

Supplement 2. Data Sharing Statement
