# Supplementary material for: First-Trimester mRNA COVID-19 Vaccination and Risk of Major Congenital Anomalies
Source: JAMA Netw Open. 2025 Oct 15;8(10):e2538039. doi: 10.1001/jamanetworkopen.2025.38039 (PMC12529215; doi:10.1001/jamanetworkopen.2025.38039)
Supplement: Supplement 1. — eTable 1. Teratogenic infections and drug use identification algorithms eTable 2. Major congenital malformations (MCMs) detection algorithms eTable 3. Maternal characteristics table with standardized mean differences (SMDs) before and after standardized mortality ratio (SMR) weighting eFigure 1. Propensity score density for the primary exposed and control groups, before (left) and after SMR weighting (right) eTable 4. Maternal and pregnancy characteristics of children depending on whether they were exposed to at least one vaccine dose during the first trimester of pregnancy (G1), or the mother received at least one vaccine dose during the second or third trimester of pregnancy (G2), or the mother received at least one dose before conception (G3), or the mother received no doses until the end of pregnancy (G4) eFigure 2. Sensitivity analyses on the main results [file jamanetwopen-e2538039-s001.pdf]

# Supplemental Online Content

Bernard C, Duchemin T, Marty L, et al. First-trimester mRNA COVID-19 vaccination and risk of major congenital anomalies. *JAMA Netw Open*. 2025;8(10):e2538039.
doi:10.1001/jamanetworkopen.2025.38039

**eTable 1.** Teratogenic infections and drug use identification algorithms
**eTable 2.** Major congenital malformations (MCMs) detection algorithms
**eTable 3.** Maternal characteristics table with standardized mean differences (SMDs) before and after standardized mortality ratio (SMR) weighting
**eFigure 1.** Propensity score density for the primary exposed and control groups, before (left) and after SMR weighting (right)
**eTable 4.** Maternal and pregnancy characteristics of children depending on whether they were exposed to at least one vaccine dose during the first trimester of pregnancy (G1), or the mother received at least one vaccine dose during the second or third trimester of pregnancy (G2), or the mother received at least one dose before conception (G3), or the mother received no doses until the end of pregnancy (G4)
**eFigure 2.** Sensitivity analyses on the main results

This supplemental material has been provided by the authors to give readers additional information about their work.

### eTable 1. Teratogenic infections and drug use identification algorithms

| TERATOGENIC INFECTIONS | | | |
|---|---|---|---|
| Infection | | Hospital discharge diagnoses codes (International Classification of Diseases, 10th revision) | Drugs (ATC class) |
| Toxoplasmosis | Mother | Toxoplasmosis (B58) | (Pyrimethamine (P01BD01) AND Sulfadiazine (J01EC02)) OR (Spiramycine (J01FA02) if at least three dispensing during pregnancy) |
| | Child | Congenital toxoplasmosis (P371) | Pyrimethamine (P01BD01) AND Sulfadiazine (J01EC02) up to one year of life |
| Syphilis | Mother | Syphilis complicating pregnancy, childbirth, and the puerperium (O981); Early syphilis (A51); Late syphilis (A52); Other and unspecified syphilis (A53); Cardiovascular syphilis (I980); Late syphilis of kidney (N290) | Benzylpenicilline (J01CE01) OR benzathine benzylpenicilline (J01CE08) |
| | Child | Congenital syphilis (A50) | Benzylpenicilline (J01CE01) OR benzathine benzylpenicilline (J01CE08) up to one year of life |
| Rubella | Mother | Rubella (B06); Rubella arthritis (M014); Maternal care for damage to fetus from maternal: cytomegalovirus/rubella (O353) | |
| | Child | Congenital rubella syndrome (P350) | |
| Cytomegalovirus | Mother | Cytomegaloviral disease (B25); Cytomegaloviral mononucleosis (B271); HIV disease resulting in Cytomegaloviral disease (B202); Cytomegaloviral cholangitis (K8700); Cytomegaloviral colitis (K93820); Cytomegaloviral retinitis (H3200); Maternal care for damage to fetus from maternal: cytomegalovirus/rubella (O353) | |
| | Child | Congenital cytomegalovirus infection (P351) | |
| Herpes | Mother | Herpes gestationis (O264), only PD/RD | |
| | Child | Congenital herpes viral [herpes simplex] infection (P352), only PD/RD | |
| Varicella | Mother | Varicella (B01) | |
| | Child | Congenital varicella (P358) | |
| Lymphocytic choriomeningitis virus | Mother | Lymphocytic choriomeningitis (A872) | |
| | Child | | |
| Zika | Mother | Zika virus disease (A925) | |
| | Child | Congenital Zika virus disease (P354) | |

- *For each diagnosis, one hospital discharge diagnosis code or one drug dispensing was sufficient to have a high sensitivity of diagnosis.*
- *All codes were used to identify diseases including PD/RD/AD during all pregnancy: PD: Principal diagnoses. RD: Related diagnoses. AD: Associated diagnoses. For herpes, only PD/RD were used as intrauterine herpes is very rare compared to neonatal herpes and the risk of low specificity is greater.*
- *Child Hospital discharge diagnoses were retrieved up to two years of life.*

| TERATOGENIC DRUGS. |
|---|

© 2025 Bernard C et al. *JAMA Network Open*.

| Anatomical Therapeutic Chemical (ATC) Classification System | Therapeutic class | Drug |
|---|---|---|
| A02BB01 | Prostaglandins | Misoprostol |
| B01AA02<br>B01AA03<br>B01AA07<br>B01AA12 | Vitamin K antagonists | Phenindione<br>Warfarin<br>Acenocoumarol<br>Fluindione |
| D05AX05 | Antipsoriatics for topical use | Tazarotene |
| D05BB02 | Antipsoriatics for systematic use | Acitretin |
| D10BA01 | Retinoids for treatment of acne | Isotretinoin |
| D11AH04 | Agents for dermatitis, oral | Alitretinoin |
| G03XA01<br>G03XC01 | Sex hormones and modulators of the genital system | Danazol<br>Raloxifene |
| G04CB01<br>G04CB02 | Drugs used in benign prostatic hypertrophy | Finasteride<br>Dutasteride |
| J05AP01 | Direct acting antivirals | Ribavirin |
| L01A | Alkylating agents | All alkylating agents |
| L01B | Antimetabolites | All antimetabolites |
| L01C | Plant alkaloids and other natural products | All plant alkaloids and other natural products |
| L01D | Cyctotoxic antibiotics and related substances | All cyctotoxic antibiotics and related substances |
| L01X | Other antineoplastic agents | All other antineoplastic agents |
| L02AB01<br>L02AE03 | Hormones and related agents | Megestrol<br>Goserelin |
| L02BA01<br>L02BA03<br>L02BB03<br>L02BG03<br>L02BG06 | Hormone antagonists and related agents | Tamoxifen<br>Fulvestrant<br>Bicalutamide<br>Anastrozole<br>Exemestane |
| L04AA06<br>L04AA13<br>L04AA31<br>L04AA40<br>L04AX02<br>L04AX03 | Immunosuppressants | Mycophenolic acid<br>Leflunomide<br>Teriflunomide<br>Cladribine<br>Thalidomide<br>Methotrexate |
| N03AG01<br>N03AX11 | Antiepileptics | Valproic acid<br>Topiramate |

© 2025 Bernard C et al. *JAMA Network Open*.

## eTable 2. Major congenital malformations (MCMs) detection algorithms

| | Identification criteria: ICD-10 diagnosis codes, exclusion criteria and medical procedures when necessary | CCAM codes used for medical procedures, surgical repair, and imaging |
|---|---|---|
| **Anomalies of the nervous system** | | |
| Anencephaly and similar malformations | Q00 | |
| Encephalocele | Q01, exclude if associated with Anencephaly (Q00) | |
| Spina Bifida | Q05, exclude if associated with Anencephaly (Q00) or Encephalocele (Q01) | |
| Congenital hydrocephalus | Q03, exclude if associated with Neural Tube defect group (Q00, Q01, Q05) | |
| Microcephaly | Q02, exclude if associated with Neural Tube defect group (Q00, Q01, Q05) + at least 1 MRI within 2 years or death | |
| Arhinencephaly/Holoprosencephaly | Q041, Q042, exclude if associated with Neural Tube defect group (Q00, Q01, Q05) | |
| Congenital malformations of corpus callosum | Q040, exclude if associated with Neural Tube defect group (Q00, Q01, Q05) | |
| **Anomalies of the eyes** | | |
| Cystic eyeball/Other anophthalmos/Microphthalmos | Q110, Q111, Q112 | |
| Cystic eyeball/Other anophthalmos | Q110, Q111 | |
| Congenital cataract | Q120 + specific medical procedures within 1 year or death | BFPA002, BGFA008, BFGA002, BGFA001, BFGA008 |
| Congenital glaucoma | Q150 + specific medical procedures within 1 year or death | BHQP002, BEFA008, BEPA003, BGFA014 |
| **Anomalies of the ear, face and neck** | | |
| Congenital absence of (ear) auricle/Congenital absence atresia and structure of auditory canal (external) | Q160, Q161 | |
| **Congenital heart defects** | | |
| Common arterial trunk | Q200 | |
| Double outlet right ventricle | Q201 | |
| Double outlet left ventricle | Q202 | |
| Discordant ventriculoarterial connection | Q203 | |
| Discordant atrioventricular connection | Q205 + surgical repair within 1 year OR death | DZMA010 |
| Double inlet ventricle | Q204, exclude if associated with hypoplastic left heart syndrome (Q234) or hypoplastic right heart syndrome (Q226) | |
| Ventricular septal defect | Q210 | |
| Atrial septal defect, incl. persistent foramen ovale | Q211 + at least one echography within 1 year OR death | DZQJ001, DZQJ006, DZQJ008, DZQJ009, DZQJ010, DZQJ011, DZQM00, DZQM006 |
| Atrioventricular septal defect | Q212 | |

© 2025 Bernard C et al. *JAMA Network Open*.

| | | |
|---|---|---|
| Tetralogy of Fallot | Q213 | |
| Congenital tricuspid stenosis | Q224 | |
| Ebstein anomaly | Q225 | |
| Congenital pulmonary valve stenosis | Q221 | |
| Pulmonary valve atresia | Q220 | |
| Congenital stenosis of aortic valve | Q230 | |
| Congenital mitral stenosis | Q232 + surgical repair within 1 year OR death | |
| Hypoplastic left heart syndrome | Q234 | |
| Hypoplastic right heart syndrome | Q226 | |
| Coarctation of aorta | Q251 | |
| Atresia of aorta | Q252 | |
| Total anomalous pulmonary venous connection | Q262 | |
| Patent ductus arteriosus | Q250 + surgical closure within 1 year OR Q25O still present after 6 months OR death AND not part of a ductus dependent congenital heart defects, namely: transposition of great arteries (Q203), hypoplastic left heart (Q234) and coarctation of aorta (Q251) | DASF001 |
| **Respiratory anomalies** | | |
| Choanal atresia | Q300+ surgical repair within 1 year or death | GCME004, GCME003, GCME002, GCMA001, GCME001, GCCD001 |
| **Oro-facial clefts** | | |
| Cleft palate | Q35 exclude if associated with holoprosencephaly, anencephaly, or cleft lip subgroups | |
| Cleft lip/Cleft palate with cleft lip | Q36, Q37 | |
| **Anomalies of the digestive system** | | |
| Atresia of oesophagus with/without tracheo-oesophageal fistula | Q390, Q391 | |
| Congenital absence, atresia and stenosis of duodenum | Q410 | |
| Congenital absence, atresia and stenosis of jejunum/ileum/ other specified parts of small intestine | Q411-Q418 | |
| Congenital absence, atresia and stenosis of anus/rectum with/without fistula | Q420-Q423 + surgical repair within 1 year or death | HJAD001, HJEA001, HJEA002, HJEA003, HJEA004, HHCA002, HJMA001, HKEA001, HKMA006, HHCC007 |
| Hirschprung disease | Q431 + surgical repair within 1 year or death | HJFD003, HJFC001, HJFA016, HHCA002, HHCC007 |
| Congenital malformations of intestinal fixation | Q433 | |
| Atresia of bile ducts | Q442 + surgical repair within 1 year or death | HLCA001 |
| Annular pancreas | Q451 | |
| Congenital diaphragmatic hernia | Q790 | |

© 2025 Bernard C et al. *JAMA Network Open*.

| | | |
|---|---|---|
| **Abdominal wall defects** | | |
| Gastroschisis | Q793+ surgical repair within 1 year or death | LMSA003, LMSA001, LMSA005 |
| Exomphalos | Q792 + surgical repair within 1 year or death | LMSA003, LMSA001, LMSA005 |
| **Congenital anomalies of kidney and urinary tract** | | |
| Renal agenesis, unilateral | Q600 | |
| Renal agenesis, bilateral/Potter syndrome | Q601, Q606 | |
| Renal dysplasia | Q614 | |
| Congenital hydronephrosis/Atresia and stenosis of ureter/Other obstructive defects of renal pelvis and ureter | Q620, Q621, Q623 exclude if associated with Q627 + at least 2 echographies within 1 year or death | JAQM003, JAQM004, JAQJ001, JAQM001 |
| Lobulated, fused and horseshoe kidney/Ectopic kidney | Q631, Q632 | |
| Epispadias/ Exstrophy of urinary bladder | Q640, Q641 | |
| Congenital posterior urethral valves | Q642 + surgical repair within 1 year or death | JEFE005, JEPH001 |
| **Genital** | | |
| Prune belly syndrome | Q794 | |
| Hypospadias | Q54 excluding Q544 + surgical repair within 1 or 2 years | JEMA006, JEMA014, JEMA019, JEMA020, JEMA021, JEMA011 |
| Indeterminate sex and pseudohermaphroditism | Q56 | |
| **Limb anomalies** | | |
| Reduction defects of upper/lower/unspecified limb | Q71, Q72, Q73 | |
| Talipes equinovarus | Q660 + specific medical procedures within 1 year | NHRP003, NJAB001, PCPB002 |
| Congenital dislocation of hip, unilateral/bilateral/unspecified | Q650, Q651, Q652 + surgical repair within 1 year OR at least 2 diagnostic tests within 1 year OR death | Surgery: NEEP003, NEEA004, ZEMP002, NEQP001, NEQH001, NEEP006, NEQP002, NZMP012, ZEMP010 diagnostic test: NEQM001, NEQH002, NAQK071, NEQC001 |
| Polydactyly | Q69 + surgical repair within 1 year OR death | MZFA008, MZFA012, MZFA015, MZFA014, NZFA011, NZFA012 |
| Syndactyly | Q70 + surgical repair within 1 year OR death | MJPA014, MZPA002, QDPA001 |
| **Other anomalies** | | |
| Craniosynostosis | Q750 + surgical repair within 1 year or death | LAFA900, LAMA006, LANC001, LAPA005, LAPA006, LAPA008, LAPA016, LAEA002, LAEA004, LAEA006, LAEA009, LARA001, LARA002, LARA003, LARA004 |

© 2025 Bernard C et al. *JAMA Network Open*.

| | |
|---|---|
| Situs inversus | Q893 |
| Septo-optic dysplasia | Q044 |
| Vascular disruption anomalies | Q411, Q412, Q418, Q710, Q712, Q720, Q722, Q730, Q793 |
| Laterality anomalies | Q206, Q240, Q890, Q893 |
| Other anomalies | Q86 |
| **Chromosomal** | |
| Skeletal dysplasia | Q77, Q780-Q788 |
| Down syndrome | Q90 |
| Trisomy 13/Patau syndrome | Q914-Q917 |
| Trisomy 18/Edwards syndrome | Q910-Q913 |
| Turner syndrome | Q96 |
| Triploidy and polyploidy | Q927 |

*ICD-10: 10th version of the International Classification of Disease*
*CCAM: classification commune des actes médicaux (French medical classification of clinical procedures)*

© 2025 Bernard C et al. *JAMA Network Open*.

**eTable 3. Maternal characteristics table with standardized mean differences (SMDs) before and after standardized mortality ratio (SMR) weighting**

| CHARACTERISTIC | Exposure to at least a dose of an mRNA COVID-19 vaccine during the first trimester of pregnancy: | | Weighting | |
|---|---|---|---|---|
| | Control, N = 397,226 (75%) | Exposed, N = 130,338 (25%) | None | SMR |
| Age class, No. (%) | | | 0.08 | 0.00 |
| *<20* | 8,598 (2.2%) | 2,209 (1.7%) | | |
| *20-24* | 51,338 (12.9%) | 14,422 (11.1%) | | |
| *25-29* | 121,374 (30.6%) | 38,808 (29.8%) | | |
| *30-34* | 133,765 (33.7%) | 46,403 (35.6%) | | |
| *35-39* | 66,929 (16.8%) | 23,379 (17.9%) | | |
| *>=40* | 15,222 (3.8%) | 5,117 (3.9%) | | |
| Social deprivation index quintiles | | | 0.07 | 0.00 |
| *Quintile 1 (Least deprived)* | 78,177 (19.7%) | 28,280 (21.7%) | | |
| *Quintile 2* | 81,035 (20.4%) | 27,221 (20.9%) | | |
| *Quintile 3* | 78,226 (19.7%) | 25,384 (19.5%) | | |
| *Quintile 4* | 76,281 (19.2%) | 24,418 (18.7%) | | |
| *Quintile 5 (Most deprived)* | 80,685 (20.3%) | 23,916 (18.3%) | | |
| *Unknown* | 2,822 (0.7%) | 1,119 (0.9%) | | |
| C2S | 68,498 (17.2%) | 15,529 (11.9%) | 0.15 | 0.00 |
| AME | 1,937 (0.5%) | 158 (0.1%) | 0.07 | 0.00 |
| Region of residence | | | 0.16 | 0.00 |
| *AUVERGNE RHONE ALPES* | 49,955 (12.6%) | 15,860 (12.2%) | | |
| *BOURGOGNE FRANCHE COMTE* | 14,725 (3.7%) | 4,975 (3.8%) | | |
| *BRETAGNE* | 17,028 (4.3%) | 7,342 (5.6%) | | |
| *CENTRE VAL DE LOIRE* | 14,726 (3.7%) | 5,104 (3.9%) | | |
| *CORSE* | 1,633 (0.4%) | 372 (0.3%) | | |
| *GRAND EST* | 30,308 (7.6%) | 9,537 (7.3%) | | |
| *HAUTS DE FRANCE* | 37,008 (9.3%) | 13,616 (10.4%) | | |
| *ILE DE FRANCE* | 91,968 (23.2%) | 27,188 (20.9%) | | |
| *NORMANDIE* | 18,541 (4.7%) | 7,564 (5.8%) | | |
| *NOUVELLE AQUITAINE* | 30,518 (7.7%) | 11,441 (8.8%) | | |
| *OCCITANIE* | 34,930 (8.8%) | 10,022 (7.7%) | | |

© 2025 Bernard C et al. *JAMA Network Open*.

| CHARACTERISTIC | Exposure to at least a dose of an mRNA COVID-19 vaccine during the first trimester of pregnancy: | | Weighting | |
|---|---|---|---|---|
| | Control, N = 397,226 (75%) | Exposed, N = 130,338 (25%) | None | SMR |
| *PAYS DE LA LOIRE* | 21,488 (5.4%) | 9,167 (7.0%) | | |
| *PROVENCE ALPES COTE D'AZUR* | 34,398 (8.7%) | 8,150 (6.3%) | | |
| Ultrasonography during T1 | 353,678 (89.0%) | 119,179 (91.4%) | -0.08 | 0.00 |
| Ultrasonography during T2 | 369,380 (93.0%) | 123,198 (94.5%) | -0.06 | 0.00 |
| Ultrasonography during T3 | 371,785 (93.6%) | 123,376 (94.7%) | -0.05 | 0.00 |
| Periconceptional folic acid dispensation | 212,477 (53.5%) | 73,981 (56.8%) | -0.07 | 0.00 |
| Number of pregnancies less than 22 weeks (since 2006) | | | 0.03 | 0.00 |
| *0* | 297,580 (74.9%) | 98,988 (75.9%) | | |
| *1* | 70,494 (17.7%) | 22,664 (17.4%) | | |
| *2 or more* | 29,152 (7.3%) | 8,686 (6.7%) | | |
| Number of pregnancies over 22 weeks (since 2006) | | | 0.04 | 0.00 |
| *1st pregnancy* | 148,582 (37.4%) | 49,029 (37.6%) | | |
| *2nd pregnancy* | 125,271 (31.5%) | 43,247 (33.2%) | | |
| *3rd pregnancy and more* | 123,373 (31.1%) | 38,062 (29.2%) | | |
| Use of Assisted Reproductive Technology | 14,515 (3.7%) | 4,411 (3.4%) | 0.01 | 0.00 |
| Confirmed Covid infection (T1) | 28,213 (7.1%) | 4,966 (3.8%) | 0.15 | 0.00 |
| Tobacco consumption | 46,753 (11.8%) | 16,125 (12.4%) | -0.02 | 0.00 |
| Alcohol consumption | 2,153 (0.5%) | 829 (0.6%) | -0.01 | 0.00 |
| Opioid consumption | 2,083 (0.5%) | 616 (0.5%) | 0.01 | 0.00 |
| Obesity | 27,805 (7.0%) | 9,723 (7.5%) | -0.02 | 0.00 |
| Pre-existing Diabetes | 3,342 (0.8%) | 1,294 (1.0%) | -0.02 | 0.00 |
| *Type 1 diabetes* | 973 (0.2%) | 409 (0.3%) | -0.01 | 0.00 |
| *Type 2 diabetes* | 1,524 (0.4%) | 575 (0.4%) | -0.01 | 0.00 |
| Hypertension | 4,373 (1.1%) | 1,650 (1.3%) | -0.02 | 0.00 |
| Stroke | 480 (0.1%) | 175 (0.1%) | 0.00 | 0.00 |
| Other Cardiovascular disease | 2,583 (0.7%) | 921 (0.7%) | -0.01 | 0.00 |
| Antidepressant use | 10,913 (2.7%) | 4,496 (3.4%) | -0.04 | 0.00 |

© 2025 Bernard C et al. *JAMA Network Open*.

| CHARACTERISTIC | Exposure to at least a dose of an mRNA COVID-19 vaccine during the first trimester of pregnancy: | | Weighting | |
|---|---|---|---|---|
| | Control, N = 397,226 (75%) | Exposed, N = 130,338 (25%) | None | SMR |
| Neuroleptic use | 1,692 (0.4%) | 715 (0.5%) | -0.02 | 0.00 |
| Anxiolytic or Hypnotic use | 8,075 (2.0%) | 3,236 (2.5%) | -0.03 | 0.00 |
| Multiple sclerosis | 736 (0.2%) | 265 (0.2%) | 0.00 | 0.00 |
| Epilepsy | 1,097 (0.3%) | 422 (0.3%) | -0.01 | 0.00 |
| Chronic respiratory disease | 9,554 (2.4%) | 3,541 (2.7%) | -0.02 | 0.00 |
| Inflammatory or skin disease | 10,407 (2.6%) | 3,576 (2.7%) | -0.01 | 0.00 |
| Cancer | 2,982 (0.8%) | 1,013 (0.8%) | 0.00 | 0.00 |
| HIV infection | 581 (0.1%) | 242 (0.2%) | -0.01 | 0.00 |

[1] n (%)

[2] Standardized Mean Difference

[3] CI = Confidence Interval

© 2025 Bernard C et al. *JAMA Network Open*.

**eFigure 1. Propensity score density for the primary exposed and control groups, before (left) and after SMR weighting (right)**

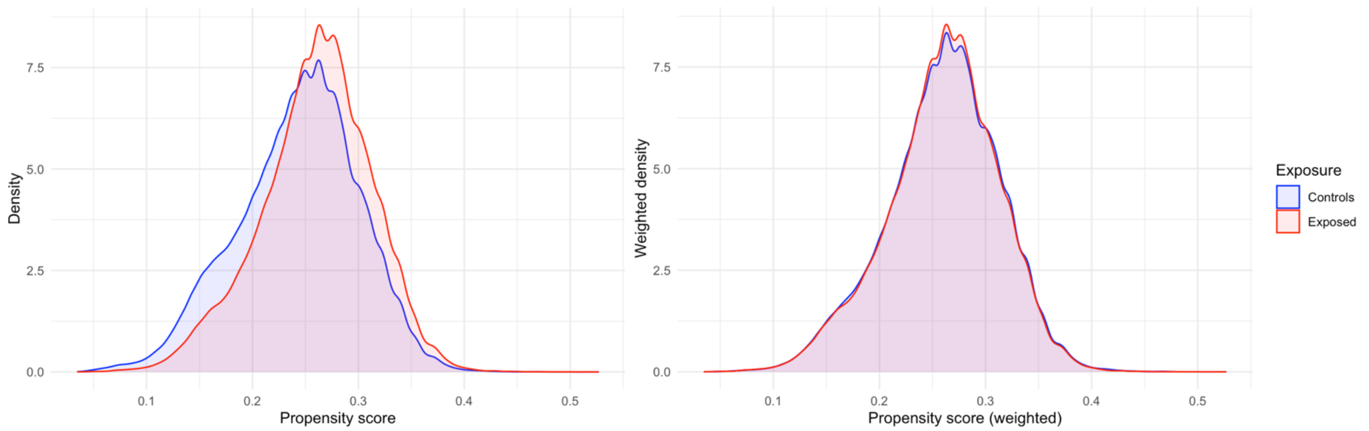


© 2025 Bernard C et al. *JAMA Network Open*.

**eTable 4. Maternal and pregnancy characteristics of children depending on whether they were exposed to at least one vaccine dose during the first trimester of pregnancy (G1), or the mother received at least one vaccine dose during the second or third trimester of pregnancy (G2), or the mother received at least one dose before conception (G3), or the mother received no doses until the end of pregnancy (G4)**

| CHARACTERISTIC | G1: Dose during T1, N = 130,338 (25%) | G2: Dose during T2 or T3, N = 123,659 (23%) | G3: Dose only before conception, N = 116,381 (22%) | G4: No dose during/before pregnancy, N = 157,186 (30%) |
|---|---|---|---|---|
| Age class, No. (%) | | | | |
| *<20* | 2,209 (1.7%) | 1,497 (1.2%) | 2,059 (1.8%) | 5,042 (3.2%) |
| *20-24* | 14,422 (11%) | 11,671 (9.4%) | 14,069 (12%) | 25,598 (16%) |
| *25-29* | 38,808 (30%) | 36,138 (29%) | 35,770 (31%) | 49,466 (31%) |
| *30-34* | 46,403 (36%) | 46,266 (37%) | 40,274 (35%) | 47,225 (30%) |
| *35-39* | 23,379 (18%) | 22,950 (19%) | 19,823 (17%) | 24,156 (15%) |
| *>=40* | 5,117 (3.9%) | 5,137 (4.2%) | 4,386 (3.8%) | 5,699 (3.6%) |
| Social deprivation index quintiles | | | | |
| *Quintile 1 (Least deprived)* | 28,280 (22%) | 29,119 (24%) | 24,126 (21%) | 24,932 (16%) |
| *Quintile 2* | 27,221 (21%) | 26,643 (22%) | 24,259 (21%) | 30,133 (19%) |
| *Quintile 3* | 25,384 (19%) | 23,663 (19%) | 22,796 (20%) | 31,767 (20%) |
| *Quintile 4* | 24,418 (19%) | 22,151 (18%) | 21,966 (19%) | 32,164 (20%) |
| *Quintile 5 (Most deprived)* | 23,916 (18%) | 21,095 (17%) | 22,399 (19%) | 37,191 (24%) |
| *Unknown* | 1,119 (0.9%) | 988 (0.8%) | 835 (0.7%) | 999 (0.6%) |
| C2S | 15,529 (12%) | 12,175 (9.8%) | 16,566 (14%) | 39,757 (25%) |
| AME | 158 (0.1%) | 153 (0.1%) | 252 (0.2%) | 1,532 (1.0%) |
| Region of residence | | | | |
| *AUVERGNE RHONE ALPES* | 15,860 (12%) | 14,888 (12%) | 15,064 (13%) | 20,003 (13%) |
| *BOURGOGNE FRANCHE COMTE* | 4,975 (3.8%) | 4,526 (3.7%) | 4,137 (3.6%) | 6,062 (3.9%) |
| *BRETAGNE* | 7,342 (5.6%) | 6,818 (5.5%) | 4,960 (4.3%) | 5,250 (3.3%) |
| *CENTRE VAL DE LOIRE* | 5,104 (3.9%) | 4,754 (3.8%) | 4,170 (3.6%) | 5,802 (3.7%) |
| *CORSE* | 372 (0.3%) | 355 (0.3%) | 494 (0.4%) | 784 (0.5%) |
| *GRAND EST* | 9,537 (7.3%) | 9,275 (7.5%) | 8,700 (7.5%) | 12,333 (7.8%) |
| *HAUTS DE FRANCE* | 13,616 (10%) | 13,267 (11%) | 10,966 (9.4%) | 12,775 (8.1%) |
| *ILE DE FRANCE* | 27,188 (21%) | 26,595 (22%) | 28,809 (25%) | 36,564 (23%) |
| *NORMANDIE* | 7,564 (5.8%) | 6,802 (5.5%) | 5,740 (4.9%) | 5,999 (3.8%) |
| *NOUVELLE AQUITAINE* | 11,441 (8.8%) | 10,500 (8.5%) | 8,556 (7.4%) | 11,462 (7.3%) |
| *OCCITANIE* | 10,022 (7.7%) | 9,647 (7.8%) | 9,651 (8.3%) | 15,632 (9.9%) |
| *PAYS DE LA LOIRE* | 9,167 (7.0%) | 8,268 (6.7%) | 6,061 (5.2%) | 7,159 (4.6%) |
| *PROVENCE ALPES COTE D AZUR* | 8,150 (6.3%) | 7,964 (6.4%) | 9,073 (7.8%) | 17,361 (11%) |
| Ultrasonography during T1 | 119,179 (91%) | 113,583 (92%) | 106,675 (92%) | 133,420 (85%) |
| Ultrasonography during T2 | 123,198 (95%) | 117,002 (95%) | 109,872 (94%) | 142,506 (91%) |
| Ultrasonography during T3 | 123,376 (95%) | 117,221 (95%) | 109,793 (94%) | 144,771 (92%) |
| Periconceptional folic acid dispensation | 73,981 (57%) | 73,249 (59%) | 66,415 (57%) | 72,813 (46%) |
| Number of pregnancies less than 22 weeks (since 2006) | | | | |

© 2025 Bernard C et al. *JAMA Network Open*.

| | | | | |
|---|---|---|---|---|
| *0* | 98,988 (76%) | 95,286 (77%) | 86,787 (75%) | 115,507 (73%) |
| *1* | 22,664 (17%) | 21,084 (17%) | 21,244 (18%) | 28,166 (18%) |
| *2 or more* | 8,686 (6.7%) | 7,289 (5.9%) | 8,350 (7.2%) | 13,513 (8.6%) |
| Number of pregnancies over 22 weeks (since 2006) | | | | |
| *1st pregnancy* | 49,029 (38%) | 48,084 (39%) | 41,816 (36%) | 58,682 (37%) |
| *2nd pregnancy* | 43,247 (33%) | 42,638 (34%) | 38,591 (33%) | 44,042 (28%) |
| *3rd pregnancy and more* | 38,062 (29%) | 32,937 (27%) | 35,974 (31%) | 54,462 (35%) |
| Use of Assisted Reproductive Technology | 4,411 (3.4%) | 6,056 (4.9%) | 4,398 (3.8%) | 4,061 (2.6%) |
| Confirmed Covid infection (T1) | 4,966 (3.8%) | 2,469 (2.0%) | 16,952 (15%) | 8,792 (5.6%) |
| Tobacco consumption | 16,125 (12%) | 13,799 (11%) | 12,753 (11%) | 20,201 (13%) |
| Alcohol consumption | 829 (0.6%) | 625 (0.5%) | 561 (0.5%) | 967 (0.6%) |
| Opioid consumption | 616 (0.5%) | 469 (0.4%) | 417 (0.4%) | 1,197 (0.8%) |
| Obesity | 9,723 (7.5%) | 8,573 (6.9%) | 8,439 (7.3%) | 10,793 (6.9%) |
| Pre-existing Diabetes | 1,294 (1.0%) | 1,150 (0.9%) | 1,049 (0.9%) | 1,143 (0.7%) |
| Hypertension | 1,650 (1.3%) | 1,646 (1.3%) | 1,387 (1.2%) | 1,340 (0.9%) |
| Stroke | 175 (0.1%) | 165 (0.1%) | 131 (0.1%) | 184 (0.1%) |
| Other Cardiovascular disease | 921 (0.7%) | 908 (0.7%) | 805 (0.7%) | 870 (0.6%) |
| Antidepressant use | 4,496 (3.4%) | 4,139 (3.3%) | 3,376 (2.9%) | 3,398 (2.2%) |
| Neuroleptic use | 715 (0.5%) | 593 (0.5%) | 473 (0.4%) | 626 (0.4%) |
| Anxiolytic or Hypnotic use | 3,236 (2.5%) | 2,759 (2.2%) | 2,400 (2.1%) | 2,916 (1.9%) |
| Multiple sclerosis | 265 (0.2%) | 254 (0.2%) | 224 (0.2%) | 258 (0.2%) |
| Epilepsy | 422 (0.3%) | 373 (0.3%) | 301 (0.3%) | 423 (0.3%) |
| Chronic respiratory disease | 3,541 (2.7%) | 3,232 (2.6%) | 2,983 (2.6%) | 3,339 (2.1%) |
| Inflammatory or skin disease | 3,576 (2.7%) | 3,382 (2.7%) | 3,238 (2.8%) | 3,787 (2.4%) |
| Cancer | 1,013 (0.8%) | 1,085 (0.9%) | 943 (0.8%) | 954 (0.6%) |
| HIV infection | 242 (0.2%) | 222 (0.2%) | 188 (0.2%) | 171 (0.1%) |

© 2025 Bernard C et al. *JAMA Network Open*.

## *eFigure 2. Sensitivity analyses on the main results*

| Type of MCM | Exposed Group n (per 10,000) | Control Group n (per 10,000) | wOR [95% CI] |
|---|---|---|---|
| **Any MCM** | | | |
| Primary analysis | 2,302/130,337 (176.6) | 7,128/397,214 (179.4) | 0.98 [0.93; 1.04] |
| Control : Excluding vaccined < 31 days before | 2,301/130,335 (176.5) | 6,048/337,496 (179.2) | 0.99 [0.93; 1.05] |
| Control : Received a vaccine dose during T2/T3 | 2,302/130,332 (176.6) | 2,261/123,659 (182.8) | 0.95 [0.90; 1.01] |
| Control : Received a vaccine dose prior to conception | 2,302/130,337 (176.6) | 2,055/116,380 (176.6) | 0.99 [0.93; 1.05] |
| Control : No dose receipt prior or during pregnancy | 2,300/130,330 (176.5) | 2,812/157,183 (178.9) | 1.00 [0.94; 1.06] |
| Exposure : Between 0 and 8 GW | 1,512/84,687 (178.5) | 7,128/397,209 (179.5) | 1.00 [0.93; 1.07] |
| Exposure : Primary vaccination series (Dose 1/2) | 1,427/80,044 (178.3) | 7,128/397,215 (179.4) | 0.99 [0.92; 1.07] |
| Exposure : Booster vaccination (Dose 3) | 875/50,292 (174.0) | 7,127/397,157 (179.5) | 0.97 [0.89; 1.07] |
| Exposure : single exposure (1 dose) | 1,932/109,761 (176.0) | 7,128/397,212 (179.5) | 0.98 [0.92; 1.05] |
| Exposure : double exposure (2 doses) | 370/20,544 (180.1) | 7,113/396,747 (179.3) | 0.98 [0.85; 1.14] |
| Weighting : Overlap approach (ATO) | 2,302/130,337 (176.6) | 7,128/397,214 (179.4) | 0.99 [0.92; 1.05] |
| Exclusion when COVID−19 during T1 | 2,204/125,371 (175.8) | 6,655/369,008 (180.3) | 0.98 [0.92; 1.04] |
| Removed Extreme PScores (1%) | 2,255/127,973 (176.2) | 6,973/389,026 (179.2) | 0.98 [0.93; 1.04] |
| **Cardiac MCM** | | | |
| Primary analysis | 760/130,337 (58.3) | 2,315/397,214 (58.3) | 1.01 [0.92; 1.12] |
| Control : Excluding vaccined < 31 days before | 759/130,335 (58.2) | 1,998/337,496 (59.2) | 1.00 [0.91; 1.11] |
| Control : Received a vaccine dose during T2/T3 | 760/130,332 (58.3) | 701/123,659 (56.7) | 1.00 [0.90; 1.10] |
| Control : Received a vaccine dose prior to conception | 760/130,337 (58.3) | 667/116,380 (57.3) | 0.99 [0.89; 1.09] |
| Control : No dose receipt prior or during pregnancy | 759/130,330 (58.2) | 947/157,183 (60.2) | 0.99 [0.90; 1.10] |
| Exposure : Between 0 and 8 GW | 501/84,687 (59.2) | 2,315/397,209 (58.3) | 1.03 [0.91; 1.17] |
| Exposure : Primary vaccination series (Dose 1/2) | 462/80,044 (57.7) | 2,315/397,215 (58.3) | 1.00 [0.88; 1.13] |
| Exposure : Booster vaccination (Dose 3) | 298/50,292 (59.3) | 2,315/397,157 (58.3) | 1.04 [0.89; 1.23] |
| Exposure : single exposure (1 dose) | 633/109,761 (57.7) | 2,315/397,212 (58.3) | 1.01 [0.90; 1.13] |
| Exposure : double exposure (2 doses) | 127/20,544 (61.8) | 2,311/396,747 (58.2) | 1.03 [0.80; 1.32] |
| Weighting : Overlap approach (ATO) | 760/130,337 (58.3) | 2,315/397,214 (58.3) | 1.01 [0.90; 1.14] |
| Exclusion when COVID−19 during T1 | 733/125,371 (58.5) | 2,168/369,008 (58.8) | 1.01 [0.91; 1.12] |
| Removed Extreme PScores (1%) | 742/127,973 (58.0) | 2,263/389,026 (58.2) | 1.01 [0.91; 1.12] |
| **Limb MCM** | | | |
| Primary analysis | 421/130,337 (32.3) | 1,188/397,214 (29.9) | 1.09 [0.95; 1.25] |
| Control : Excluding vaccined < 31 days before | 421/130,335 (32.3) | 1,002/337,496 (29.7) | 1.10 [0.96; 1.26] |
| Control : Received a vaccine dose during T2/T3 | 421/130,332 (32.3) | 352/123,659 (28.5) | 1.13 [0.98; 1.30] |
| Control : Received a vaccine dose prior to conception | 421/130,337 (32.3) | 359/116,380 (30.8) | 1.05 [0.92; 1.21] |
| Control : No dose receipt prior or during pregnancy | 421/130,330 (32.3) | 477/157,183 (30.3) | 1.08 [0.94; 1.24] |
| Exposure : Between 0 and 8 GW | 279/84,687 (32.9) | 1,188/397,209 (29.9) | 1.11 [0.93; 1.31] |
| Exposure : Primary vaccination series (Dose 1/2) | 272/80,044 (34.0) | 1,188/397,215 (29.9) | 1.14 [0.95; 1.35] |
| Exposure : Booster vaccination (Dose 3) | 149/50,292 (29.6) | 1,188/397,157 (29.9) | 1.01 [0.80; 1.26] |
| Exposure : single exposure (1 dose) | 352/109,761 (32.1) | 1,188/397,212 (29.9) | 1.08 [0.93; 1.26] |
| Exposure : double exposure (2 doses) | 69/20,544 (33.6) | 1,185/396,747 (29.9) | 1.11 [0.79; 1.57] |
| Weighting : Overlap approach (ATO) | 421/130,337 (32.3) | 1,188/397,214 (29.9) | 1.09 [0.93; 1.28] |
| Exclusion when COVID−19 during T1 | 407/125,371 (32.5) | 1,113/369,008 (30.2) | 1.09 [0.94; 1.25] |
| Removed Extreme PScores (1%) | 414/127,973 (32.4) | 1,161/389,026 (29.8) | 1.10 [0.96; 1.27] |
| **Kidney or urinary tract MCM** | | | |
| Primary analysis | 304/130,337 (23.3) | 1,018/397,214 (25.6) | 0.88 [0.76; 1.03] |
| Control : Excluding vaccined < 31 days before | 304/130,335 (23.3) | 853/337,496 (25.3) | 0.90 [0.77; 1.05] |
| Control : Received a vaccine dose during T2/T3 | 304/130,332 (23.3) | 343/123,659 (27.7) | 0.85 [0.73; 0.99] |
| Control : Received a vaccine dose prior to conception | 304/130,337 (23.3) | 298/116,380 (25.6) | 0.89 [0.76; 1.04] |
| Control : No dose receipt prior or during pregnancy | 304/130,330 (23.3) | 377/157,183 (24.0) | 0.92 [0.79; 1.08] |
| Exposure : Between 0 and 8 GW | 189/84,687 (22.3) | 1,018/397,209 (25.6) | 0.85 [0.70; 1.03] |
| Exposure : Primary vaccination series (Dose 1/2) | 184/80,044 (23.0) | 1,018/397,215 (25.6) | 0.88 [0.72; 1.07] |
| Exposure : Booster vaccination (Dose 3) | 120/50,292 (23.9) | 1,018/397,157 (25.6) | 0.89 [0.70; 1.14] |
| Exposure : single exposure (1 dose) | 255/109,761 (23.2) | 1,018/397,212 (25.6) | 0.88 [0.74; 1.04] |
| Exposure : double exposure (2 doses) | 49/20,544 (23.9) | 1,017/396,747 (25.6) | 0.91 [0.62; 1.34] |
| Weighting : Overlap approach (ATO) | 304/130,337 (23.3) | 1,018/397,214 (25.6) | 0.89 [0.75; 1.07] |
| Exclusion when COVID−19 during T1 | 289/125,371 (23.1) | 944/369,008 (25.6) | 0.87 [0.74; 1.02] |
| Removed Extreme PScores (1%) | 300/127,973 (23.4) | 995/389,026 (25.6) | 0.88 [0.76; 1.03] |
| **Genital MCM** | | | |
| Primary analysis | 242/130,337 (18.6) | 791/397,214 (19.9) | 0.91 [0.77; 1.09] |
| Control : Excluding vaccined < 31 days before | 242/130,335 (18.6) | 658/337,496 (19.5) | 0.93 [0.78; 1.10] |
| Control : Received a vaccine dose during T2/T3 | 242/130,332 (18.6) | 275/123,659 (22.2) | 0.85 [0.71; 1.00] |
| Control : Received a vaccine dose prior to conception | 242/130,337 (18.6) | 228/116,380 (19.6) | 0.96 [0.80; 1.15] |
| Control : No dose receipt prior or during pregnancy | 242/130,330 (18.6) | 288/157,183 (18.3) | 0.99 [0.83; 1.18] |
| Exposure : Between 0 and 8 GW | 153/84,687 (18.1) | 791/397,209 (19.9) | 0.90 [0.72; 1.11] |
| Exposure : Primary vaccination series (Dose 1/2) | 154/80,044 (19.2) | 791/397,215 (19.9) | 0.96 [0.77; 1.19] |
| Exposure : Booster vaccination (Dose 3) | 88/50,292 (17.5) | 791/397,157 (19.9) | 0.85 [0.64; 1.13] |
| Exposure : single exposure (1 dose) | 214/109,761 (19.5) | 791/397,212 (19.9) | 0.95 [0.79; 1.15] |
| Exposure : double exposure (2 doses) | 28/20,544 (13.6) | 791/396,747 (19.9) | 0.69 [0.42; 1.11] |
| Weighting : Overlap approach (ATO) | 242/130,337 (18.6) | 791/397,214 (19.9) | 0.91 [0.75; 1.12] |
| Exclusion when COVID−19 during T1 | 225/125,371 (17.9) | 736/369,008 (19.9) | 0.88 [0.74; 1.06] |
| Removed Extreme PScores (1%) | 237/127,973 (18.5) | 780/389,026 (20.1) | 0.91 [0.76; 1.08] |

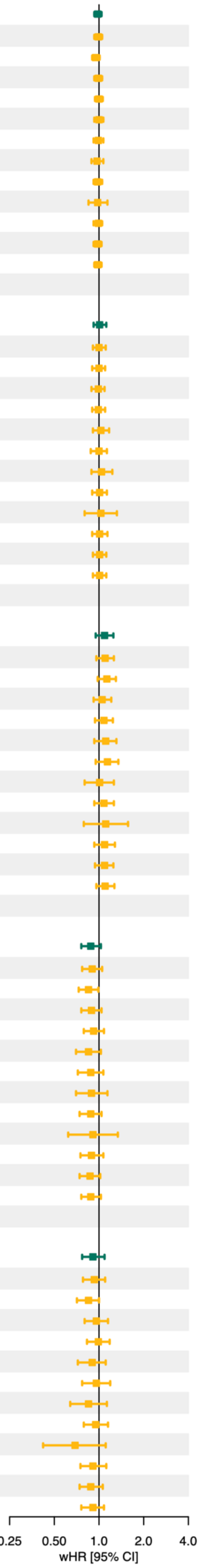


© 2025 Bernard C et al. *JAMA Network Open*.

| Type of MCM | Exposed Group n (per 10,000) | Control Group n (per 10,000) | wOR [95% CI] |
|---|---|---|---|
| **Digestive system MCM** | | | |
| Primary analysis | 151/130,337 (11.6) | 551/397,214 (13.9) | 0.84 [0.68; 1.04] |
| Control : Excluding vaccined < 31 days before | 151/130,335 (11.6) | 474/337,496 (14.0) | 0.82 [0.66; 1.02] |
| Control : Received a vaccine dose during T2/T3 | 151/130,332 (11.6) | 171/123,659 (13.8) | 0.84 [0.68; 1.05] |
| Control : Received a vaccine dose prior to conception | 151/130,337 (11.6) | 142/116,380 (12.2) | 0.89 [0.72; 1.11] |
| Control : No dose receipt prior or during pregnancy | 151/130,330 (11.6) | 238/157,183 (15.1) | 0.78 [0.63; 0.96] |
| Exposure : Between 0 and 8 GW | 97/84,687 (11.5) | 551/397,209 (13.9) | 0.83 [0.64; 1.09] |
| Exposure : Primary vaccination series (Dose 1/2) | 86/80,044 (10.7) | 551/397,215 (13.9) | 0.78 [0.59; 1.03] |
| Exposure : Booster vaccination (Dose 3) | 65/50,292 (12.9) | 550/397,157 (13.8) | 0.95 [0.67; 1.33] |
| Exposure : single exposure (1 dose) | 123/109,761 (11.2) | 551/397,212 (13.9) | 0.81 [0.64; 1.03] |
| Exposure : double exposure (2 doses) | 28/20,544 (13.6) | 548/396,747 (13.8) | 0.99 [0.58; 1.66] |
| Weighting : Overlap approach (ATO) | 151/130,337 (11.6) | 551/397,214 (13.9) | 0.83 [0.65; 1.07] |
| Exclusion when COVID−19 during T1 | 143/125,371 (11.4) | 521/369,008 (14.1) | 0.82 [0.66; 1.02] |
| Removed Extreme PScores (1%) | 145/127,973 (11.3) | 538/389,026 (13.8) | 0.82 [0.66; 1.02] |
| **Cleft lip or palate** | | | |
| Primary analysis | 169/130,337 (13.0) | 505/397,214 (12.7) | 1.00 [0.81; 1.24] |
| Control : Excluding vaccined < 31 days before | 169/130,335 (13.0) | 426/337,496 (12.6) | 1.00 [0.81; 1.24] |
| Control : Received a vaccine dose during T2/T3 | 169/130,332 (13.0) | 183/123,659 (14.8) | 0.87 [0.71; 1.07] |
| Control : Received a vaccine dose prior to conception | 169/130,337 (13.0) | 146/116,380 (12.5) | 1.06 [0.85; 1.31] |
| Control : No dose receipt prior or during pregnancy | 169/130,330 (13.0) | 176/157,183 (11.2) | 1.20 [0.96; 1.50] |
| Exposure : Between 0 and 8 GW | 113/84,687 (13.3) | 505/397,209 (12.7) | 1.03 [0.79; 1.34] |
| Exposure : Primary vaccination series (Dose 1/2) | 101/80,044 (12.6) | 505/397,215 (12.7) | 0.98 [0.75; 1.29] |
| Exposure : Booster vaccination (Dose 3) | 68/50,292 (13.5) | 505/397,157 (12.7) | 1.04 [0.74; 1.46] |
| Exposure : single exposure (1 dose) | 144/109,761 (13.1) | 505/397,212 (12.7) | 1.02 [0.81; 1.28] |
| Exposure : double exposure (2 doses) | 25/20,544 (12.2) | 504/396,747 (12.7) | 0.92 [0.54; 1.59] |
| Weighting : Overlap approach (ATO) | 169/130,337 (13.0) | 505/397,214 (12.7) | 1.01 [0.78; 1.29] |
| Exclusion when COVID−19 during T1 | 165/125,371 (13.2) | 467/369,008 (12.7) | 1.02 [0.82; 1.27] |
| Removed Extreme PScores (1%) | 167/127,973 (13.0) | 494/389,026 (12.7) | 1.01 [0.81; 1.25] |
| **Nervous system MCM** | | | |
| Primary analysis | 161/130,337 (12.4) | 499/397,214 (12.6) | 1.04 [0.83; 1.29] |
| Control : Excluding vaccined < 31 days before | 161/130,335 (12.4) | 425/337,496 (12.6) | 1.04 [0.84; 1.30] |
| Control : Received a vaccine dose during T2/T3 | 161/130,332 (12.4) | 155/123,659 (12.5) | 0.95 [0.77; 1.18] |
| Control : Received a vaccine dose prior to conception | 161/130,337 (12.4) | 139/116,380 (11.9) | 1.02 [0.82; 1.27] |
| Control : No dose receipt prior or during pregnancy | 160/130,330 (12.3) | 205/157,183 (13.0) | 1.06 [0.85; 1.33] |
| Exposure : Between 0 and 8 GW | 111/84,687 (13.1) | 499/397,209 (12.6) | 1.08 [0.83; 1.42] |
| Exposure : Primary vaccination series (Dose 1/2) | 105/80,044 (13.1) | 499/397,215 (12.6) | 1.07 [0.81; 1.41] |
| Exposure : Booster vaccination (Dose 3) | 56/50,292 (11.1) | 499/397,157 (12.6) | 0.98 [0.68; 1.41] |
| Exposure : single exposure (1 dose) | 127/109,761 (11.6) | 499/397,212 (12.6) | 0.99 [0.77; 1.26] |
| Exposure : double exposure (2 doses) | 34/20,544 (16.5) | 498/396,747 (12.6) | 1.28 [0.77; 2.12] |
| Weighting : Overlap approach (ATO) | 161/130,337 (12.4) | 499/397,214 (12.6) | 1.03 [0.80; 1.33] |
| Exclusion when COVID−19 during T1 | 155/125,371 (12.4) | 472/369,008 (12.8) | 1.03 [0.82; 1.29] |
| Removed Extreme PScores (1%) | 156/127,973 (12.2) | 486/389,026 (12.5) | 1.03 [0.82; 1.29] |
| **Chromosomal anomaly** | | | |
| Primary analysis | 93/130,337 (7.1) | 359/397,214 (9.0) | 0.85 [0.65; 1.12] |
| Control : Excluding vaccined < 31 days before | 93/130,335 (7.1) | 313/337,496 (9.3) | 0.83 [0.63; 1.10] |
| Control : Received a vaccine dose during T2/T3 | 93/130,332 (7.1) | 82/123,659 (6.6) | 1.04 [0.78; 1.39] |
| Control : Received a vaccine dose prior to conception | 93/130,337 (7.1) | 102/116,380 (8.8) | 0.84 [0.64; 1.11] |
| Control : No dose receipt prior or during pregnancy | 93/130,330 (7.1) | 175/157,183 (11.1) | 0.69 [0.53; 0.90] |
| Exposure : Between 0 and 8 GW | 63/84,687 (7.4) | 359/397,209 (9.0) | 0.88 [0.62; 1.23] |
| Exposure : Primary vaccination series (Dose 1/2) | 59/80,044 (7.4) | 359/397,215 (9.0) | 0.85 [0.60; 1.20] |
| Exposure : Booster vaccination (Dose 3) | 34/50,292 (6.8) | 359/397,157 (9.0) | 0.86 [0.54; 1.36] |
| Exposure : single exposure (1 dose) | 75/109,761 (6.8) | 359/397,212 (9.0) | 0.83 [0.61; 1.13] |
| Exposure : double exposure (2 doses) | 18/20,544 (8.8) | 359/396,747 (9.0) | 0.97 [0.51; 1.85] |
| Weighting : Overlap approach (ATO) | 93/130,337 (7.1) | 359/397,214 (9.0) | 0.85 [0.62; 1.17] |
| Exclusion when COVID−19 during T1 | 90/125,371 (7.2) | 330/369,008 (8.9) | 0.87 [0.65; 1.15] |
| Removed Extreme PScores (1%) | 91/127,973 (7.1) | 349/389,026 (9.0) | 0.85 [0.64; 1.13] |
| **Eye MCM** | | | |
| Primary analysis | 42/130,337 (3.2) | 114/397,214 (2.9) | 1.17 [0.75; 1.83] |
| Control : Excluding vaccined < 31 days before | 42/130,335 (3.2) | 96/337,496 (2.8) | 1.24 [0.79; 1.96] |
| Control : Received a vaccine dose during T2/T3 | 42/130,332 (3.2) | 33/123,659 (2.7) | 1.18 [0.75; 1.84] |
| Control : Received a vaccine dose prior to conception | 42/130,337 (3.2) | 28/116,380 (2.4) | 1.28 [0.81; 2.03] |
| Control : No dose receipt prior or during pregnancy | 42/130,330 (3.2) | 53/157,183 (3.4) | 0.98 [0.64; 1.50] |
| Exposure : Between 0 and 8 GW | 24/84,687 (2.8) | 114/397,209 (2.9) | 1.03 [0.58; 1.82] |
| Exposure : Primary vaccination series (Dose 1/2) | 30/80,044 (3.7) | 114/397,215 (2.9) | 1.36 [0.78; 2.35] |
| Exposure : Booster vaccination (Dose 3) | 12/50,292 (2.4) | 114/397,157 (2.9) | 0.87 [0.40; 1.90] |
| Exposure : single exposure (1 dose) | 35/109,761 (3.2) | 114/397,212 (2.9) | 1.17 [0.72; 1.90] |
| Exposure : double exposure (2 doses) | 7/20,544 (3.4) | 113/396,747 (2.8) | 1.21 [0.40; 3.63] |
| Weighting : Overlap approach (ATO) | 42/130,337 (3.2) | 114/397,214 (2.9) | 1.17 [0.70; 1.96] |
| Exclusion when COVID−19 during T1 | 41/125,371 (3.3) | 106/369,008 (2.9) | 1.19 [0.76; 1.87] |
| Removed Extreme PScores (1%) | 41/127,973 (3.2) | 112/389,026 (2.9) | 1.15 [0.73; 1.80] |

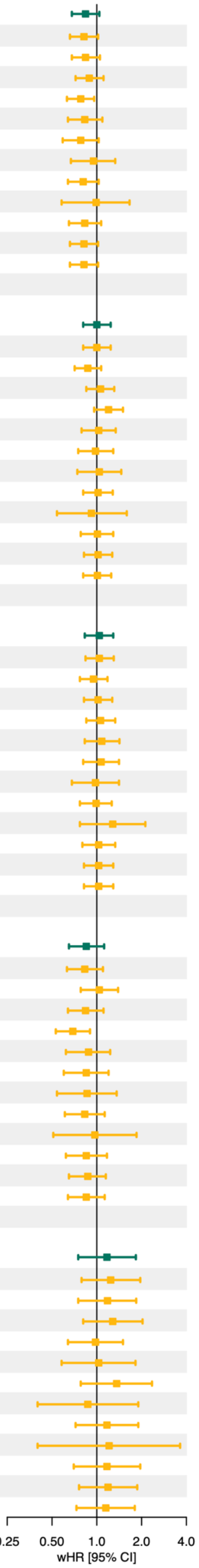


© 202

| Type of MCM | Exposed Group n (per 10,000) | Control Group n (per 10,000) | wOR [95% CI] |
|---|---|---|---|
| **Abdominal wall MCM** | | | |
| Primary analysis | 39/130,337 (3.0) | 100/397,214 (2.5) | 1.20 [0.75; 1.91] |
| Control : Excluding vaccined < 31 days before | 39/130,335 (3.0) | 86/337,496 (2.5) | 1.18 [0.74; 1.88] |
| Control : Received a vaccine dose during T2/T3 | 39/130,332 (3.0) | 38/123,659 (3.1) | 0.90 [0.58; 1.38] |
| Control : Received a vaccine dose prior to conception | 39/130,337 (3.0) | 21/116,380 (1.8) | 1.41 [0.87; 2.30] |
| Control : No dose receipt prior or during pregnancy | 39/130,330 (3.0) | 41/157,183 (2.6) | 1.29 [0.80; 2.07] |
| Exposure : Between 0 and 8 GW | 23/84,687 (2.7) | 100/397,209 (2.5) | 1.08 [0.60; 1.95] |
| Exposure : Primary vaccination series (Dose 1/2) | 23/80,044 (2.9) | 100/397,215 (2.5) | 1.13 [0.62; 2.05] |
| Exposure : Booster vaccination (Dose 3) | 16/50,292 (3.2) | 100/397,157 (2.5) | 1.33 [0.63; 2.80] |
| Exposure : single exposure (1 dose) | 33/109,761 (3.0) | 100/397,212 (2.5) | 1.22 [0.74; 2.03] |
| Exposure : double exposure (2 doses) | 6/20,544 (2.9) | 100/396,747 (2.5) | 1.09 [0.34; 3.48] |
| Weighting : Overlap approach (ATO) | 39/130,337 (3.0) | 100/397,214 (2.5) | 1.20 [0.70; 2.06] |
| Exclusion when COVID−19 during T1 | 39/125,371 (3.1) | 97/369,008 (2.6) | 1.22 [0.76; 1.94] |
| Removed Extreme PScores (1%) | 39/127,973 (3.0) | 100/389,026 (2.6) | 1.20 [0.75; 1.91] |
| **Respiratory system MCM** | | | |
| Primary analysis | 6/130,337 (0.5) | 18/397,214 (0.5) | 1.00 [0.32; 3.12] |
| Control : Excluding vaccined < 31 days before | 6/130,335 (0.5) | 16/337,496 (0.5) | 0.96 [0.31; 2.92] |
| Control : Received a vaccine dose during T2/T3 | 6/130,332 (0.5) | 4/123,659 (0.3) | 1.39 [0.40; 4.80] |
| Control : Received a vaccine dose prior to conception | 6/130,337 (0.5) | 5/116,380 (0.4) | 0.97 [0.31; 2.96] |
| Control : No dose receipt prior or during pregnancy | 6/130,330 (0.5) | 9/157,183 (0.6) | 0.72 [0.25; 2.06] |
| Exposure : Between 0 and 8 GW | 5/84,687 (0.6) | 18/397,209 (0.5) | 1.26 [0.34; 4.70] |
| Exposure : Primary vaccination series (Dose 1/2) | 2/80,044 (0.2) | 18/397,215 (0.5) | 0.52 [0.09; 2.90] |
| Exposure : Booster vaccination (Dose 3) | 4/50,292 (0.8) | 18/397,157 (0.5) | 1.86 [0.35; 9.72] |
| Exposure : single exposure (1 dose) | 6/109,761 (0.5) | 18/397,212 (0.5) | 1.21 [0.37; 3.97] |
| Exposure : double exposure (2 doses) | 0/20,544 (0.0) | 18/396,747 (0.5) | 0.00 [0.00; Inf] |
| Weighting : Overlap approach (ATO) | 6/130,337 (0.5) | 18/397,214 (0.5) | 0.99 [0.27; 3.73] |
| Exclusion when COVID−19 during T1 | 6/125,371 (0.5) | 18/369,008 (0.5) | 1.00 [0.32; 3.12] |
| Removed Extreme PScores (1%) | 6/127,973 (0.5) | 17/389,026 (0.4) | 1.01 [0.33; 3.16] |
| **Ear, face or neck MCM** | | | |
| Primary analysis | 5/130,337 (0.4) | 12/397,214 (0.3) | 1.14 [0.32; 4.09] |
| Control : Excluding vaccined < 31 days before | 5/130,335 (0.4) | 12/337,496 (0.4) | 0.95 [0.28; 3.21] |
| Control : Received a vaccine dose during T2/T3 | 5/130,332 (0.4) | 3/123,659 (0.2) | 1.52 [0.38; 6.10] |
| Control : Received a vaccine dose prior to conception | 5/130,337 (0.4) | 2/116,380 (0.2) | 1.62 [0.39; 6.68] |
| Control : No dose receipt prior or during pregnancy | 5/130,330 (0.4) | 7/157,183 (0.4) | 0.77 [0.24; 2.46] |
| Exposure : Between 0 and 8 GW | 4/84,687 (0.5) | 12/397,209 (0.3) | 1.42 [0.31; 6.49] |
| Exposure : Primary vaccination series (Dose 1/2) | 4/80,044 (0.5) | 12/397,215 (0.3) | 1.57 [0.33; 7.54] |
| Exposure : Booster vaccination (Dose 3) | 1/50,292 (0.2) | 12/397,157 (0.3) | 0.55 [0.05; 6.26] |
| Exposure : single exposure (1 dose) | 4/109,761 (0.4) | 12/397,212 (0.3) | 1.09 [0.26; 4.47] |
| Exposure : double exposure (2 doses) | 1/20,544 (0.5) | 12/396,747 (0.3) | 1.41 [0.07; 29.46] |
| Weighting : Overlap approach (ATO) | 5/130,337 (0.4) | 12/397,214 (0.3) | 1.19 [0.27; 5.30] |
| Exclusion when COVID−19 during T1 | 5/125,371 (0.4) | 11/369,008 (0.3) | 1.19 [0.33; 4.36] |
| Removed Extreme PScores (1%) | 5/127,973 (0.4) | 12/389,026 (0.3) | 1.14 [0.32; 4.09] |
| **Other MCM** | | | |
| Primary analysis | 123/130,337 (9.4) | 427/397,214 (10.7) | 0.89 [0.70; 1.14] |
| Control : Excluding vaccined < 31 days before | 123/130,335 (9.4) | 361/337,496 (10.7) | 0.90 [0.71; 1.15] |
| Control : Received a vaccine dose during T2/T3 | 123/130,332 (9.4) | 140/123,659 (11.3) | 0.80 [0.63; 1.01] |
| Control : Received a vaccine dose prior to conception | 123/130,337 (9.4) | 118/116,380 (10.1) | 0.95 [0.74; 1.22] |
| Control : No dose receipt prior or during pregnancy | 123/130,330 (9.4) | 169/157,183 (10.8) | 0.93 [0.73; 1.19] |
| Exposure : Between 0 and 8 GW | 85/84,687 (10.0) | 427/397,209 (10.8) | 0.95 [0.71; 1.28] |
| Exposure : Primary vaccination series (Dose 1/2) | 80/80,044 (10.0) | 427/397,215 (10.7) | 0.94 [0.70; 1.28] |
| Exposure : Booster vaccination (Dose 3) | 43/50,292 (8.6) | 427/397,157 (10.8) | 0.82 [0.55; 1.22] |
| Exposure : single exposure (1 dose) | 103/109,761 (9.4) | 427/397,212 (10.7) | 0.90 [0.69; 1.17] |
| Exposure : double exposure (2 doses) | 20/20,544 (9.7) | 425/396,747 (10.7) | 0.88 [0.48; 1.61] |
| Weighting : Overlap approach (ATO) | 123/130,337 (9.4) | 427/397,214 (10.7) | 0.90 [0.68; 1.19] |
| Exclusion when COVID−19 during T1 | 113/125,371 (9.0) | 395/369,008 (10.7) | 0.86 [0.67; 1.10] |
| Removed Extreme PScores (1%) | 122/127,973 (9.5) | 421/389,026 (10.8) | 0.90 [0.71; 1.15] |

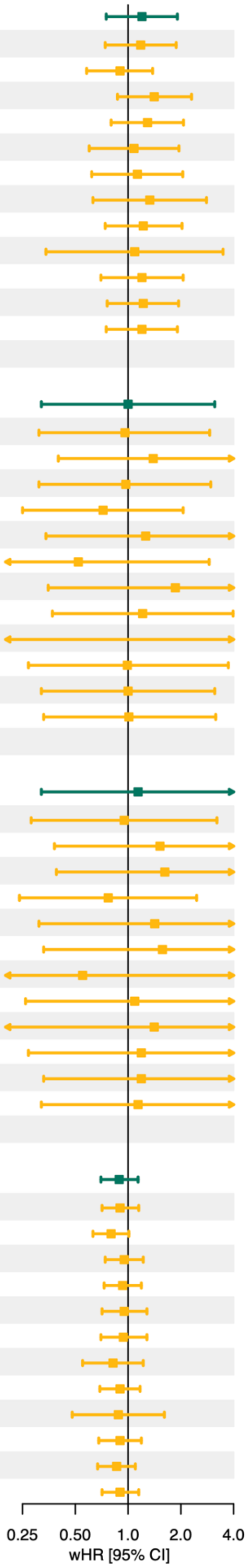


© 2025 Bernard C et al. *JAMA Network Open*.
