# Supplementary material for: First-Trimester mRNA COVID-19 Vaccination and Risk of Major Congenital Anomalies
Source: JAMA Netw Open. 2025 Oct 15;8(10):e2538039. doi: 10.1001/jamanetworkopen.2025.38039 (PMC12529215; doi:10.1001/jamanetworkopen.2025.38039)
Supplement: Supplement 2. — Data Sharing Statement [file jamanetwopen-e2538039-s002.pdf]

Data Sharing Statement

Bernard. First-Trimester mRNA COVID-19 Vaccination and Risk of Major Congenital Anomalies. *JAMA Netw Open*. Published October 15, 2025. doi:10.1001/jamanetworkopen.2025.38039

Data
Data available: No
